# Gut community structure as a risk factor for infection in *Klebsiella pneumoniae*-colonized patients

**DOI:** 10.1128/msystems.00786-24

**Published:** 2024-07-08

**Authors:** Jay Vornhagen, Krishna Rao, Michael A. Bachman

**Affiliations:** 1 Department of Microbiology & Immunology, Indiana University School of Medicine, Indianapolis, Indiana, USA; 2 Department of Internal Medicine/Infectious Diseases Division, Michigan Medicine, University of Michigan, Ann Arbor, Michigan, USA; 3 Department of Pathology, Michigan Medicine, University of Michigan, Ann Arbor, Michigan, USA; 4 Department of Microbiology & Immunology, Michigan Medicine, University of Michigan, Ann Arbor, Michigan, USA; Argonne National Laboratory, Lemont, Illinois, USA

**Keywords:** *Klebsiella*, microbiome

## Abstract

The primary risk factor for infection with members of the *Klebsiella pneumoniae* species complex is prior gut colonization, and infection is often caused by the colonizing strain. Despite the importance of the gut as a reservoir for infectious *K. pneumoniae*, little is known about the association between the gut microbiome and infection. To explore this relationship, we undertook a case-control study comparing the gut community structure of *K. pneumoniae*-colonized intensive care and hematology/oncology patients. Cases were *K. pneumoniae*-colonized patients infected by their colonizing strain (*N* = 83). Controls were *K. pneumoniae*-colonized patients who remained asymptomatic (*N* = 149). First, we characterized the gut community structure of *K. pneumoniae*-colonized patients agnostic to case status. Next, we determined that gut community data is useful for classifying cases and controls using machine learning models and that the gut community structure differed between cases and controls. *K. pneumoniae* relative abundance, a known risk factor for infection, had the greatest feature importance, but other gut microbes were also informative. Finally, we show that integration of gut community structure with bacterial genotype data enhanced the ability of machine learning models to discriminate cases and controls. Interestingly, inclusion of patient clinical variables failed to improve the ability of machine learning models to discriminate cases and controls. This study demonstrates that including gut community data with *K. pneumoniae*-derived biomarkers improves our ability to classify infection in *K. pneumoniae*-colonized patients.

**IMPORTANCE:**

Colonization is generally the first step in pathogenesis for bacteria with pathogenic potential. This step provides a unique window for intervention since a given potential pathogen has yet to cause damage to its host. Moreover, intervention during the colonization stage may help alleviate the burden of therapy failure as antimicrobial resistance rises. Yet, to understand the therapeutic potential of interventions that target colonization, we must first understand the biology of colonization and if biomarkers at the colonization stage can be used to stratify infection risk. The bacterial genus *Klebsiella* includes many species with varying degrees of pathogenic potential. Members of the *K. pneumoniae* species complex have the highest pathogenic potential. Patients colonized in their gut by these bacteria are at higher risk of subsequent infection with their colonizing strain. However, we do not understand if other members of the gut microbiota can be used as a biomarker to predict infection risk. In this study, we show that the gut microbiota differs between colonized patients who develop an infection versus those who do not. Additionally, we show that integrating gut microbiota data with bacterial factors improves the ability to classify infections. Surprisingly, patient clinical factors were not useful for classifying infections alone or when added to microbiota-based models. This indicates that the bacterial genotype and the microbial community in which it exists may determine the progression to infection. As we continue to explore colonization as an intervention point to prevent infections in individuals colonized by potential pathogens, we must develop effective means for predicting and stratifying infection risk.

## INTRODUCTION

The gut is a vast ecosystem populated by trillions of bacteria, viruses, and microbial eukaryotes. The majority of these microbes have beneficial or neutral impacts on host health; however, some are potential pathogens. Under specific circumstances, some gut microbes can escape to distant body sites, leading to infection. One such group of pathogens is the *Klebsiella pneumoniae* species complex (referred to as “*K. pneumoniae*”). This complex contains several potentially pathogenic species of *Klebsiella*, including *K. pneumoniae*, *K. variicola*, *K. quasipneumoniae*, *K. quasivariicola* sp. nov., and *K. africana* [reviewed in reference (1)]. These bacteria are common causes of bacteremia, pneumonia, and urinary tract infection (UTI). The genome content of a given strain of *K. pneumoniae* determines its infectious potential, where the presence of virulence and fitness factors permits and enhances infectivity, and antimicrobial resistance genes complicate infection treatment (2). As of 2019, *K. pneumoniae* is the third leading global cause of death attributable to, or associated with, antimicrobial resistance (3). More research is necessary to understand *K. pneumoniae* pathogenesis. Such research may lead to improved diagnosis and treatment, and therein reduce the burden of *K. pneumoniae* disease.

*K. pneumoniae-*colonized patients are at increased risk for subsequent infection (4–6). Though few patient-centered studies determine the specific origin of infectious *K. pneumoniae*, those that have demonstrated that *K. pneumoniae-*colonized patients are infected with their colonizing strains in about ~80% of cases (4, 6, 7). Additionally, gut dominance by *K. pneumoniae* is a risk factor for infection in *K. pneumoniae-*colonized patients (8–10). The identification and interrogation of factors that permit, enhance, or restrict *K. pneumoniae* gut colonization are receiving increased attention due to the clear importance of the gut as a reservoir for infectious *K. pneumoniae*. Recent laboratory-based studies have identified novel gut fitness factors (11–14), microbes that enhance colonization resistance (15, 16), and gut community structures that are permissive or restrictive to colonization (11, 17). Despite increased interest, studies aiming to understand gut ecology in *K. pneumoniae-*colonized patients are comparatively sparse, limiting the translatability of laboratory-based findings to real-world settings.

Previously, we performed a cohort study of over 1,900 *K*. *pneumoniae*-colonized patients in the intensive care and hematology/oncology units (7). The goal of this study was to identify patient variables associated with infection. Additionally, two corresponding nested case-control studies were performed to assess the role of gut dominance in *K. pneumoniae* infection (8) and to rigorously identify infection-associated *K. pneumoniae* factors (18). Here, we aimed to leverage this case-control cohort of patients to understand the gut ecology of *K. pneumoniae-*colonized patients and determine if gut community structure can improve infection classification in *K. pneumoniae*-colonized patients. We found that community structure can improve infection classification. Machine learning models trained using gut community structure data in tandem with *K. pneumoniae* genotype can discriminate *K. pneumoniae*-colonized patients that proceed to infection from those that remain asymptomatically colonized. Additionally, this suggests that there are biologically meaningful interactions between *K. pneumoniae* and the gut microbes that dictate the outcome of colonization.

## RESULTS

### Description of study population

Two hundred and thirty-eight patients were originally selected from a cohort of 1,978 *K*. *pneumoniae*-colonized intensive care and hematology/oncology patients (7) for a nested case-control study to assess the role of gut colonization density as a risk factor for *K. pneumoniae* infection (8). Cohort identification, enrollment, clinical data extraction, chart review, case definitions, and case-control matching criteria are described in detail elsewhere (7, 8, 18). Briefly, residual rectal swabs originally collected for vancomycin-resistant *Enterococcus* screening upon admission to the intensive care units and the oncology wards from May 2017 to September 2018 at the University of Michigan hospitals in Ann Arbor, MI were used. Swabs were collected sequentially as they were being processed and without regard to any characteristics other than availability and yield. Patients were enrolled in our larger cohort study if *K. pneumoniae* was isolated from their rectal swab (7). Up to three isolates were archived from each swab along with all *K. pneumoniae* isolates from subsequent clinical cultures. Patient *K. pneumoniae* clinical cultures were evaluated as potential cases for alignment with clinical definitions for infection (7). Then, Sanger sequencing of the *wzi* locus was performed on the clinical and rectal isolates of confirmed infections to determine if the clinical isolate was concordant with the colonizing strain in the same patient (7, 8, 18). For the present study, we selected 232 patients (83 cases, 149 controls, Table 1) from the previous study based on inclusion in our previous comparative genomics study and available DNA extracted from the rectal swab most proximal to the infection (18). Cases were defined as colonized patients who met clinical criteria for concordant infection with a *K. pneumoniae* strain that was detectable in the gut prior to infection (7). The most common infection type was bacteremia, followed by UTI and respiratory infection (Table 1). Controls were defined as asymptomatically colonized patients with a negative clinical culture collected of the same type as that of the matching case. Cases were matched with controls based on rectal swab collection date, age, and sex (18). Rectal swab DNA was extracted from concordant infections and matched controls (8). 16S rRNA gene sequencing was performed using the method described by Kozich et al. (19).

**TABLE 1:** Select patient demographics

Variable		Case (*N* = 83)	Control (*N* = 149)	*P* value^*a*^
Age	Mean ± SD	60 ± 13	59 ± 12	0.556
Sex	Male	43 (51.8%)	76 (51.0%)	1.000
	Female	40 (48.2%)	71 (47.7%)	
	Missing	0 (0%)	2 (1.3%)	
Race/ethnicity	African American	10 (12.0%)	19 (12.8%)	1.000
	Asian	2 (2.4%)	1 (0.7%)	0.292
	Caucasian	70 (84.3%)	119 (79.9%)	0.482
	Hispanic or Latino	1 (1.2%)	0 (0%)	0.358
	Non-Hispanic or Latino	68 (81.9%)	116 (77.8%)	0.503
	Refused or missing	1 (1.2%)	3 (2.0%)	1.000
	Other	1 (1.2%)	5 (3.4%)	0.425
	Hispanic or Latino	0 (0%)	1 (0.7%)	1.000
	Non-Hispanic or Latino	1 (1.2%)	4 (2.7%)	0.657
	Missing	0 (0%)	5 (3.4%)	0.163
Infection site	Blood	41 (49.4%)		
	Respiratory	19 (22.9%)		
	Urine	23 (27.7%)		

^*a*^ Age: Student’s *t* test; sex/race/ethnicity: Fisher’s exact test.

### The variation in gut community structure of *K. pneumoniae*-colonized patients is shaped by the relative abundances of *Escherichia*, *Klebsiella*, and anaerobes

**Fig 1:**
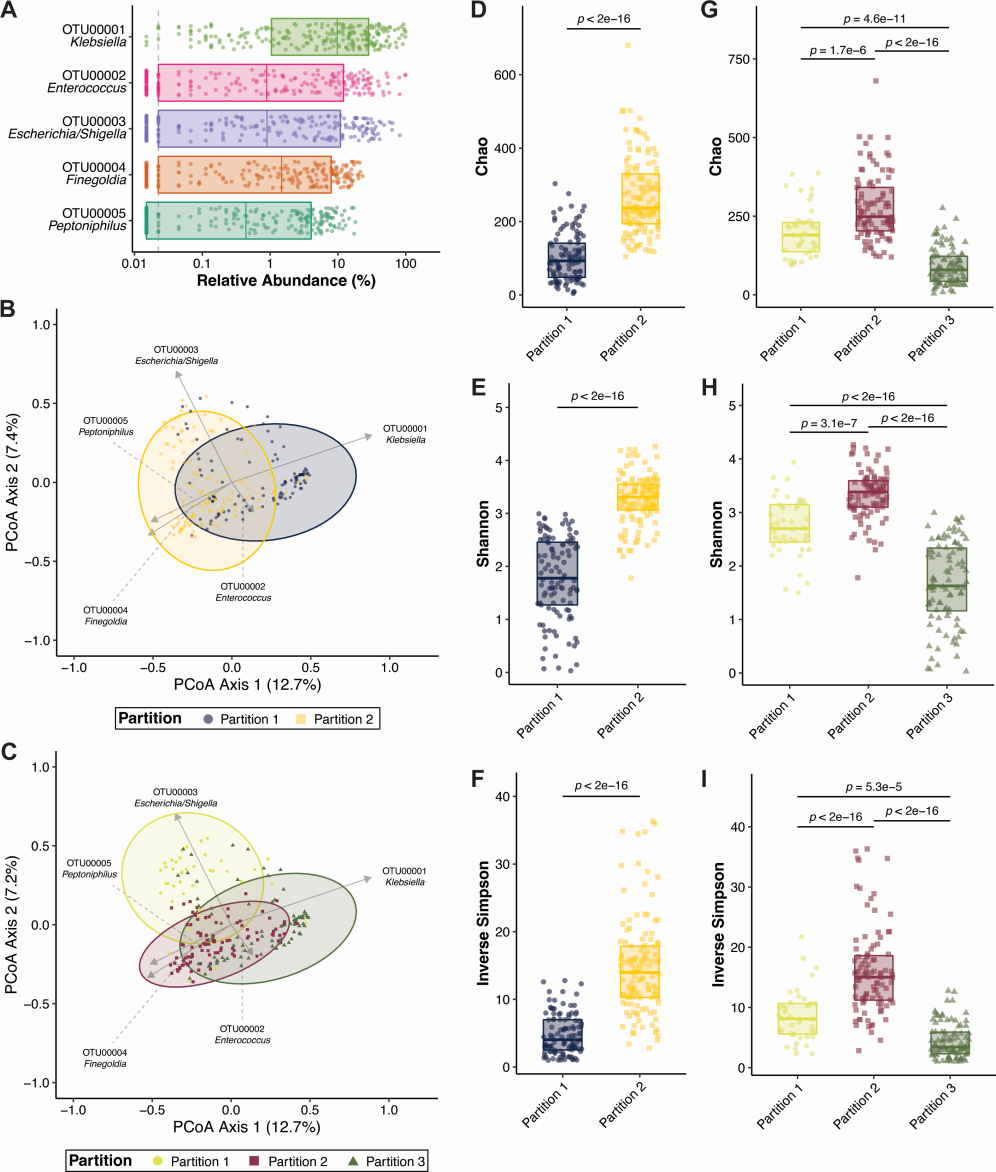
*Klebsiella pneumoniae* is the dominant gut microbe in *K. pneumoniae-*colonized patients. (**A**) Top five operational taxonomic units (OTUs) in *K. pneumoniae-*colonized patients (*N* = 232). Principal coordinates analysis with overlayed biplots of OTUs of two- (**B**) and three-partition (**C**) community clustering using Dirichlet multinomial mixtures. Analysis of the Chao, Shannon, and Inverse Simpson alpha-diversity indices in two- (**D–F**) and three-partition community clustering (G-I, boxplot indicates median with interquartile range, *P* indicates Student’s *t* test *P* value after Benjamini and Hochberg correction for multiple comparisons). For all panels, each data point indicates one patient.

First, we aimed to explore the gut community structure of *K. pneumoniae-*colonized patients agnostic of case status. *Klebsiella*, *Enterococcus*, *Escherichia/Shigella*, *Finegoldia*, and *Peptoniphilus* were the dominant gut microbiota in this study population (Fig. 1A). Probabilistic modeling using Dirichlet multinomial mixtures (20) was used to determine if metacommunities exist in our study population. The optimal number of community clusters was two (Laplace approximation = 194,340.97, Table S1), though one- and three-community clusters yielded similar fits (one-community Laplace approximation = 194,864.80, three-community Laplace approximation = 200,401.69, Table S1). Case status was not associated with metacommunity structure in either the two- [Partition 1 vs Partition 2: odds ratio (95% CI) =1.35 (0.789–2.32)] or three-community models [Partition 1 vs Partition 2: odds ratio (95% CI) =1.11 (0.505–2.46), Partition 1 vs Partition 3: odds ratio (95% CI) =0.921 (0.414–2.05), Partition 2 vs Partition 3: odds ratio (95% CI) =0.826 (0.46–1.48)]. Principal coordinates analysis revealed that OTU00001 *Klebsiella* and OTU00002 *Enterococcus* were strong components determining metacommunity structure in both two- (Partition 1, Fig. 1B) and three-partition communities (Partition 3, Fig. 1C), whereas other dominant gut microbiota influence different metacommunities (Fig. 1B and C). Alpha-diversity analysis of these metacommunities revealed that OTU00001 *Klebsiella* influenced partitions (Partition 1 and Partition 3 in two- and three-partition communities, respectively) were significantly less rich (Chao), even (Shannon) and diverse (Inverse Simpson) than other metacommunities (Fig. 1D through I). Interestingly, Partition 1 of the three-partition community clustering, which is heavily influenced by OTU00003 *Escherichia/Shigella* (Fig. 1C), was significantly less rich, even, and diverse than Partition 2 (Fig. 1G through I), which is influenced by OTU00004 *Finegoldia* and OTU00005 *Peptoniphilus* (Fig. 1C). Given that OTU00004 *Finegoldia* and OTU00005 *Peptoniphilus* are strict anaerobes and OTU00001 *Klebsiella*, OTU00002 *Enterococcus*, and OTU00003 *Escherichia/Shigella* are facultative anaerobes, it may be the case that alpha diversity is driven by the presence or absence of anaerobic bacteria in the gut in this patient population. Collectively, these data indicate that *K. pneumoniae* is the dominant gut microbe in this population of *K. pneumoniae-*colonized patients, and is associated with reduced richness, evenness, and diversity.

### Models using ASVs performed best at classifying cases and controls

**Fig 2:**
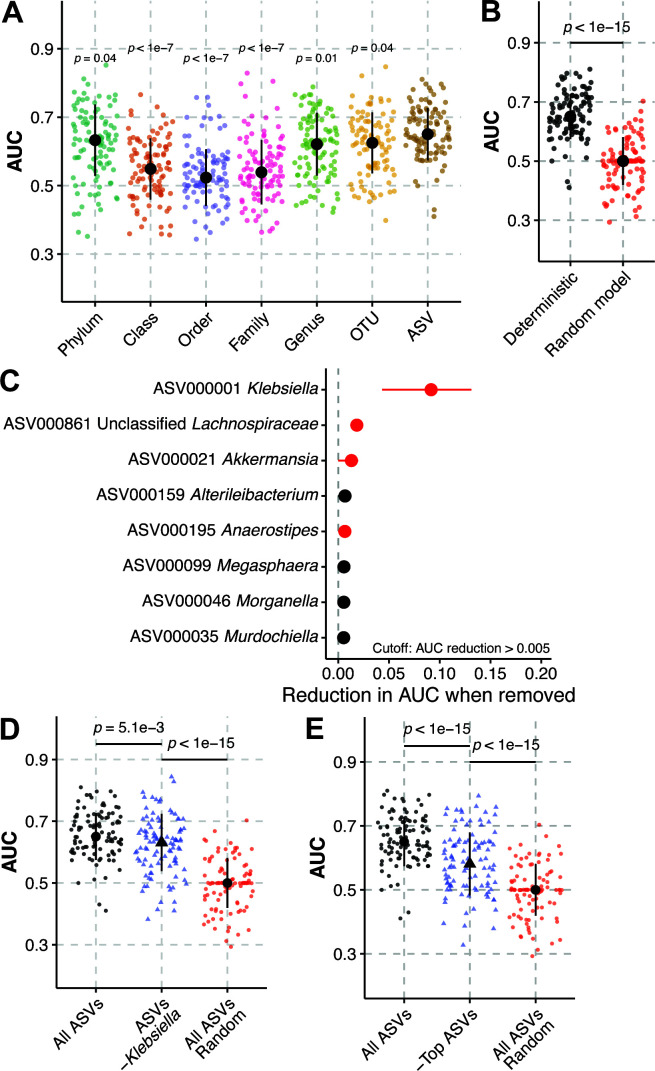
Amplicon sequence variants best discriminate cases and controls. Regularized logistic regression model performance, as measured by area under the receiver-operator characteristic curve (A, AUC) on 100 test data sets consisting of a random subset of samples (80%) to predict case status in *K. pneumoniae*-colonized patients using different taxonomical data inputs. All 83 cases and 83 randomly selected controls were used for each model. (**B**) Model performance, as measured by AUC, using ASVs as input data were compared to models where case and control status was randomized. (**C**) Top model features for regularized logistic regression models using amplicon sequence variants (ASVs) as input data “ASVs.” Circles indicate mean feature importance and lines indicate interquartile range. Feature importance values in red and black indicate a regression weight that is weighted toward cases and controls, respectively. (**D**) Model performance, as measured by AUC, using ASVs as input data compared to a model where all *Klebsiella* ASVs were omitted “ASV*s -Klebsiella*”. (**E**) Regularized logistic regression model performance on test data sets for 100 seeds predicting case status in *K. pneumoniae*-colonized patients using all ASVs (All ASVs) or excluding ASVs ASV000001, ASV000021, and ASV000816 (“-Top ASVs”). Black circles indicate median values, black lines indicate standard deviation, and *P* indicates Tukey multiple pairwise-comparison *P* value following one-way ANOVA compared to “ASV” (**A, D, E**) or Student’s *t* test *P* value (**B**). For panels (A, B, C, and D), each data point indicates one test data set.

Our next goal was to determine the ability of microbiota composition to discriminate cases from controls. To this end, we used supervised machine learning models to classify case status, using different taxonomic levels as input data. Prior to machine learning, the case and control data set was balanced (*N* = 83) by randomly selecting 83 controls and all 83 cases for each model iteration. Due to their high interpretability compared to other methods, we chose to use regularized logistic regression. To ensure optimal model performance, training was iterated across several combinations of hyperparameters [as in reference (21)], wherein the hyperparameter combination that yielded peak training performance was used as the final model (Fig. S1). This process was repeated for phylum, class, order, family, genus, operational taxonomical units binned at 97% sequence similarity (OTU), and amplicon sequence variant (ASV) level data. ASVs provided the most robust discrimination of cases and controls, followed by OTU and phylum (Fig. 2A). Additionally, models using ASVs as their input variables were most likely to yield an AUC >0.5, indicating that the classification of cases and controls was better than random chance. Other model performance metrics yielded comparable results (Table 2). Deterministic elastic net models trained on ASV data significantly outperformed models where case and control status were randomized (Fig. 2B). As we observed optimal model performance with ASVs, we decided to use the taxonomic level for further study analyses.

**TABLE 2:** Taxon level elastic net performance data^*b*^

	Phylum	Class	Order	Family	Genus	OTU	ASV
AUC**^*a*^**	**0.62 (0.55–0.7**)	**0.55 (0.48–0.61**)	**0.54 (0.5–0.58**)	**0.55 (0.5–0.6**)	**0.61 (0.55–0.69**)	**0.62 (0.55–0.68**)	0.66 (0.61–0.71)
pRAUC**^*a*^**	0.58 (0.52–0.64)	**0.52 (0.48–0.58**)	**0.48 (0.47–0.54**)	**0.51 (0.47–0.55**)	0.58 (0.51–0.63)	0.58 (0.52–0.62)	0.6 (0.55–0.64)
Accuracy	**0.59 (0.53–0.66**)	**0.52 (0.47–0.57**)	**0.52 (0.47–0.56**)	**0.54 (0.5–0.59**)	**0.58 (0.53–0.66**)	**0.59 (0.53–0.66**)	0.63 (0.59–0.69)
Sensitivity	0.6 (0.5–0.69)	0.54 (0.48–0.63)	0.5 (0.44–0.63)	0.52 (0.44–0.63)	0.57 (0.5–0.69)	0.62 (0.55–0.69)	0.55 (0.44–0.63)
Specificity	**0.57 (0.5–0.69**)	**0.51 (0.44–0.58**)	**0.54 (0.44–0.56**)	**0.57 (0.44–0.63**)	**0.59 (0.5–0.69**)	**0.57 (0.5–0.69**)	0.71 (0.63–0.75)
PPV**^*a*^**	**0.59 (0.53–0.65**)	**0.52 (0.47–0.57**)	**0.52 (0.47–0.56**)	**0.55 (0.5–0.6**)	**0.59 (0.53–0.64**)	**0.59 (0.53–0.65**)	0.66 (0.6–0.73)
NPV**^*a*^**	0.59 (0.53–0.67)	**0.52 (0.47–0.58**)	**0.52 (0.47–0.56**)	**0.54 (0.5–0.59**)	0.59 (0.53–0.64)	0.6 (0.53–0.67)	0.62 (0.57–0.67)

^*a*^ AUC: area under the receiver-operating characteristic curve; PRAUC: area under the precision-recall curve; PPV: positive predictive value; NPV: negative predictive value.

^*b*^ Median values and interquartile range are shown. Bold values are significantly different (*P* < 0.05) from ASVs by Tukey multiple pairwise-comparison *P* value following one-way ANOVA. Tukey multiple pairwise-comparison *P* value following one-way ANOVA for all comparisons can be found in Table S5.

### Classification does not solely rely on the top ASVs such as ASV000001 *Klebsiella*

Consistent with previous observations that gut dominance by *K. pneumoniae* is a risk factor for infection in colonized patients (8–10), ASV000001 *Klebsiella* was the most important feature in our regularized logistic regression models and was weighted toward cases (Fig. 2C). Interestingly, two other ASVs, ASV000021 *Akkermansia* and ASV000861 Unclassified *Lachnospiraceae*, were also highly important features weighted toward cases (Fig. 2C). This suggests that other members of the gut microbiota have discriminatory power for case status, rather than discriminatory power being limited to ASV000001 *Klebsiella*. Given the relatively high feature importance of these ASVs compared to other important features (Fig. 2C), we hypothesized that removal of the *Klebsiella* ASVs or ASVs with high feature importance may result in a model with no ability to classify cases and controls (AUC ≤0.5). Removal of all *Klebsiella* ASVs (Fig. 2D) or a combination of ASV000001, ASV000021, and ASV000861 (Fig. 2E) significantly reduced model performance; however, most models were still able to classify cases and controls better than chance (AUC >0.5) and outperformed a model where case status was randomly assigned. Similar results were observed upon removal of just ASV000001 *Klebsiella* (Fig. S2). This indicates that peak model performance does not rely solely on ASV000001, *Klebsiella*, or the three most important ASV features.

### Case and control gut community profiles differ

**Fig 3:**
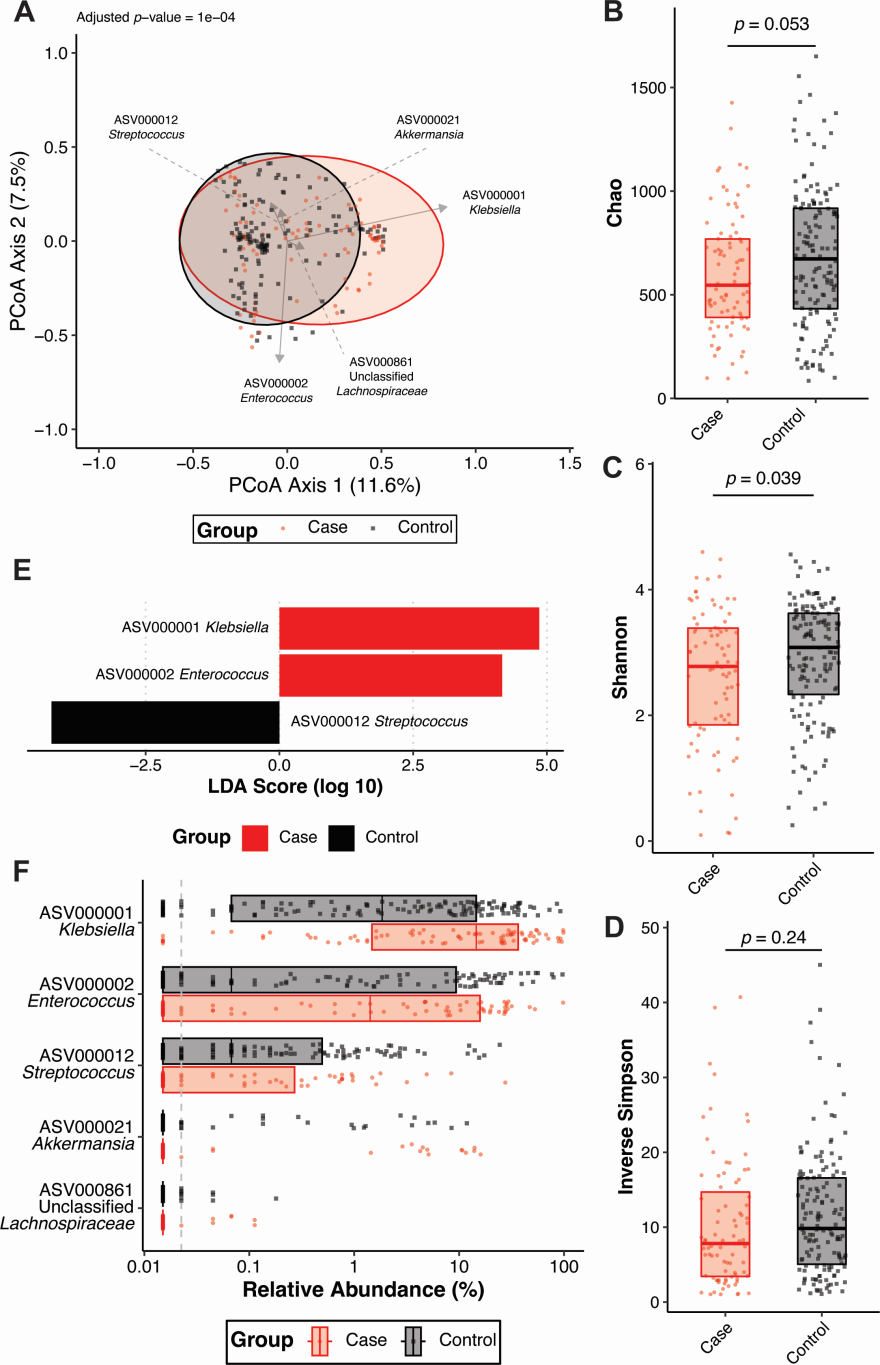
Cases and controls have distinct gut community profiles based on ASVs (**A**) Principal coordinates analysis with overlayed biplots of specific ASVs. Permutational multivariate analysis of variance (PERMANOVA, 1,000 permutations) based on the Yue and Clayton *θ* dissimilarity index was used to assess the difference in beta-diversity between cases (*N* = 83) and controls (*N* = 149). Analysis of the (**B**) Chao, (**C**) Shannon, and (**D**) Inverse Simpson alpha-diversity indices between cases (*N* = 83) and controls (*N* = 149, boxplot indicates median with interquartile range, *P* indicates Student’s *t* test *P* value). (**E**) Linear discriminant analysis (LDA) effect size was used to identify differentially abundant (*P* value < 0.05) ASVs between cases (*N* = 83) and controls (*N* = 149). (**F**) Summary of relative abundances of ASVs that were differentially abundant (Fig. 3E) between cases (*N* = 83) and controls (*N* = 149) or highly important features for classification of cases and controls using regularized logistic regression shown in Fig. 2C (boxplot indicates median with interquartile range). For all panels, each data point indicates one patient.

Given that cases and controls can be distinguished based on ASVs using machine learning, we next wanted to determine if the gut community profile of cases and controls differ. To this end, Yue and Clayton *θ* dissimilarity index was calculated for each patient and used to assess the difference in beta-diversity between cases and controls. Visualization of distances using principal coordinates analysis revealed subtly different clustering of these groups (Fig. 3A). Though variance between the two groups was highly dimensional, as indicated by the low axis loadings (Fig. 3A), the gut microbiota of cases and controls was significantly different (adjusted *P*-value = 1 × 10^−4^, Permutational multivariate analysis of variance, 1,000 permutations). Only community evenness (Shannon) was significantly different between cases and controls, though community richness and diversity displayed similar trends (Fig. 3B through D). Interestingly, the ASVs that were highly important for classifying cases and controls using machine learning models (Fig. 2B) partially differed from those enriched in either cases or controls. Linear discriminant analysis revealed that, as expected, ASV000001 *Klebsiella* was significantly enriched in cases, though unlike what was observed in the machine learning models, ASV000002 *Enterococcus* was also enriched in cases and ASV000012 *Streptococcus* was enriched in controls (Fig. 3E and F). Similar results were yielded using OTUs instead of ASVs to differentiate cases and controls (Fig. S3). Network analysis revealed that the gut community of controls was more connected than the gut community of cases (Fig. S4), suggesting a more stable gut community. Collectively, these data indicate that significant differences, not limited to *K. pneumoniae* relative abundance, exist between cases and controls that underpin the ability to discriminate these two groups based on gut community profile.

Previously, we detected the presence of multiple *K. pneumoniae* strains in colonized patients (18). A deeper exploration of ASVs revealed 30 ASVs that were classified as *Klebsiella*, and another 10,470 ASVs that were only classified to the level of Enterobacteriaceae. The majority (83.1%, 193/232) of patients had only one detectable *Klebsiella* ASV; however, 9.9% (23/323) of patients had multiple *Klebsiella* ASVs and 6.9% (16/232) had no *Klebsiella* ASVs (Fig. S5A) despite microbiological confirmation of *Klebsiella* colonization. The most abundant *Klebsiella* ASV was ASV000001, followed by ASV000019 (Fig. S5B). Importantly, only these two *Klebsiella* ASVs were included in the classification models in this study, as the rare *Klebsiella* ASVs were removed in the data preprocessing step prior to model training due to their near-zero variance between cases and controls. ASV000001 is 100% identical to the V4 region of all sequenced colonizing *K. pneumoniae* isolates in our original WGS study, except one, which had a contig break at 219 bp of the 253 bp V4 amplicon. Five samples in our data set with no detectable ASV000001 or ASV000019 had a detectable rare *Klebsiella* ASV (≤2 reads). Given that all sequenced colonizing *K. pneumoniae* isolates had V4 amplicons identical to ASV000001, very low-abundance ASVs may be present due to sequencing errors; however, it is difficult to attribute rare ASVs to sequencing error with a high degree of confidence as only a single *K. pneumoniae* strain was sequenced from each colonized patient and co-colonization does occur. Interestingly, we detected ASV000019 *Klebsiella* in several controls, though no cases (Fig. S5B). Though ASVs do not provide high-confidence species-level resolution, it was notable that the ASV000019 16S rRNA gene sequence primarily aligned to members of the *K. oxytoca* complex (22), whereas the ASV000001 16S rRNA gene sequence primarily aligned to members of the *K. pneumoniae* complex (Table S2). Interestingly, ASV000001 is absent from patients colonized by ASV000019 (Fig. S5C). Collectively, these results suggest that species-level measurement of cocolonization may be possible through targeted genomic sequencing to understand colonization dynamics of high-abundance taxa, though it may not be possible at the strain-level with current approaches. More sophisticated sequencing techniques will need to be developed to assay colonization dynamics using discarded rectal swabs.

### Inclusion of gut microbiota data with *K. pneumoniae* genotype data enhances discrimination of cases and controls

**Fig 4:**
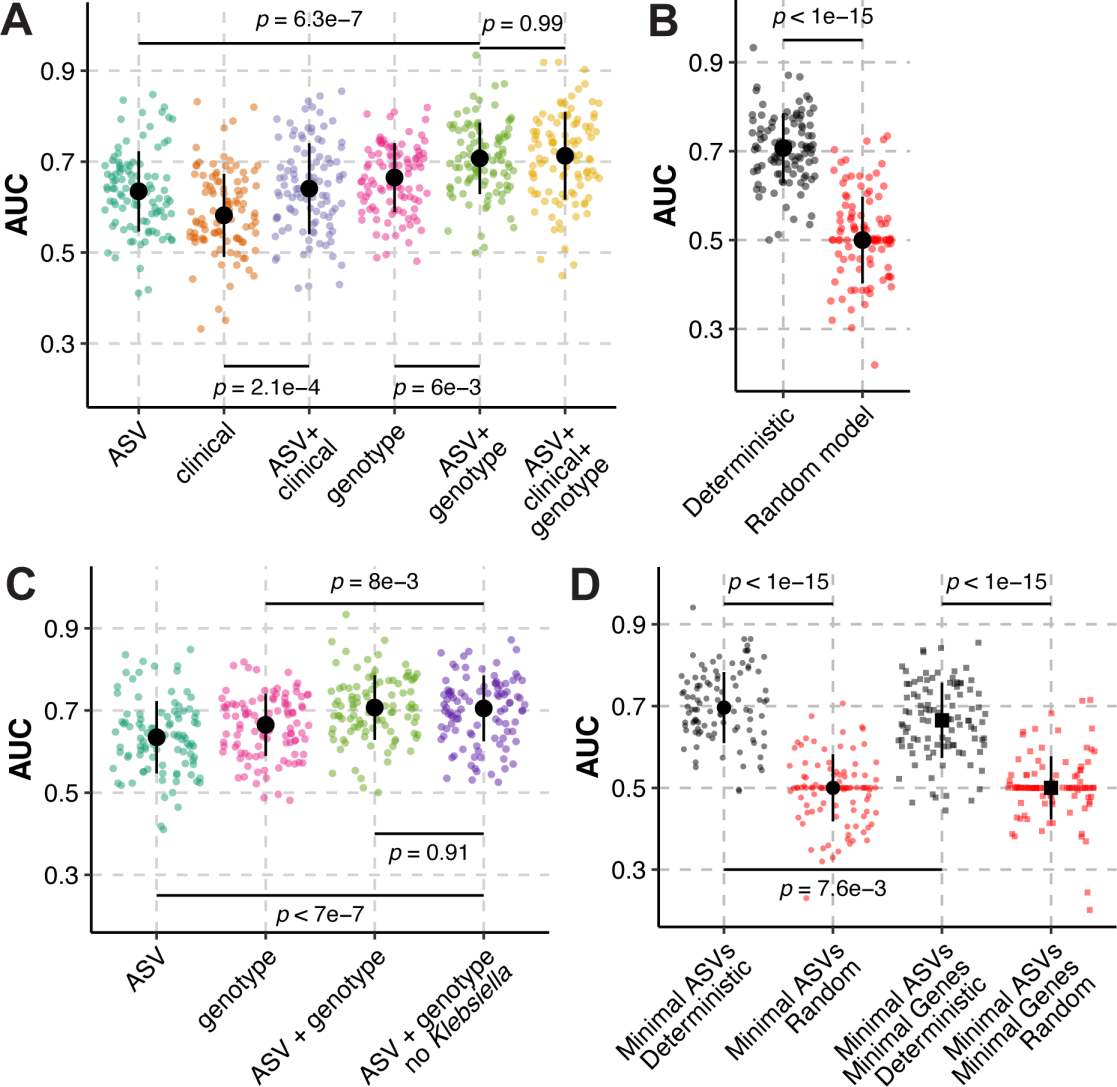
Inclusion of ASVs enhances the ability to discriminate cases from controls. Regularized logistic regression model performance, as measured by area under the receiver-operator characteristic curve (A, AUC) on 100 test data sets consisting of a random subset of samples (80%) to classify case status in *K. pneumoniae*-colonized patients using combinations of clinical variables, *K. pneumoniae* genotype, and ASVs. (**B**) Model performance, as measured by AUC, using ASV and *K. pneumoniae* genotype as input data were compared to models where case and control status was randomized. (**C**) Model performance, as measured by AUC, using combinations of ASV and *K. pneumoniae* genotype as input data were compared to models where *Klebsiella* ASV000001 and ASV000019 were omitted “ASV + genotype no *Klebsiella*.” (**D**) Regularized logistic regression model performance, as measured by AUC, on 100 test data sets using ASV000001, ASV000002, ASV000012, ASV000021, ASV000861, and *K. pneumoniae* genotype (Minimal ASV) or using ASV000001, ASV000002, ASV000012, ASV000021, and ASV000861, and the five validated *K. pneumoniae* genes from reference (18) (Minimal ASV + Minimal Genes) as data inputs. Black circles indicate median values, black lines indicate standard deviation, and *P* indicates Tukey multiple pairwise-comparison *P* value following one-way ANOVA (**A, C, D**) or Student’s *t* test *P* value (**B**). For all panels, each data point indicates one patient.

Finally, we hypothesized that the inclusion of 16S rRNA gene sequencing data with clinical factors and *K. pneumoniae* genotype would enhance the ability of machine learning models to discriminate cases and controls. To test this hypothesis, we permutated ASVs with patient factors and *K. pneumoniae* genotype in our regularized logistic regression models. Eighty-four clinical factors, including several laboratory values, antibiotic exposure, and comorbidities, were included (Table S3) and the 27 infection-associated genes identified in our previous comparative genomics study were included as *K. pneumoniae* genotype (18). Clinical data were missing for two patients, so these patients were excluded from these analyses. Use of clinical factors as the sole input variables led to poor model performance (Table 3; Fig. 4A): 14/100 of regularized logistic regression models have an AUC ≤0.5. Addition of ASVs to clinical factors enhanced median model performance (Fig. 4A). The lack of classifying ability of the clinical factors, especially antimicrobial exposure is somewhat surprising, as gut dominance is a known risk factor for infection (8–10), and disruption of the gut microbiota, such as what occurs with antibiotic exposure, leads to dominance in experimental gut colonization models (11, 13). Therefore, one may expect that antibiotic exposure would be an important feature for discriminating cases and controls in this study. Rather, exposure to most antibiotics was not among the most important features in regularized logistic regression models using clinical factors as the input variables (Fig. S6A) and the effects of antibiotic exposure on model performance was negligible (Fig. S6B). This included a variable for “high-risk” antibiotic exposure, which is a composite variable that includes β-lactam/β-lactamase inhibitor combinations, carbapenems, third- and fourth-generation cephalosporins, fluoroquinolones, clindamycin, and oral vancomycin based on their impact on indigenous gut microbiota (23). The only antibiotic present among the most important features was aminoglycoside exposure, and its effects on model performance were subtle (Fig. S6A). The importance of antibiotics was further reduced when ASVs were included (Figure S6C and D).

**TABLE 3:** ASV, clinical variable, and *K. pneumoniae* genotype elastic net performance data^*b*^

	ASV	Clinical	Genotype	ASV + clinical	ASV + genotype	ASV + clinical + genotype
AUC**^*a*^**	0.66 (0.61–0.71)	**0.59 (0.53–0.66**)	0.66 (0.61–0.72)	0.64 (0.58–0.71)	**0.71 (0.66–0.76**)	**0.71 (0.64–0.78**)
prAUC**^*a*^**	0.6 (0.55–0.64)	0.55 (0.5–0.6)	**0.53 (0.48–0.57**)	0.6 (0.54–0.65)	**0.65 (0.61–0.7**)	**0.66 (0.6–0.72**)
Accuracy	0.63 (0.59–0.69)	**0.56 (0.5–0.6**)	0.63 (0.56–0.66)	0.6 (0.53–0.66)	**0.65 (0.59–0.69**)	**0.65 (0.59–0.69**)
Sensitivity	0.55 (0.44–0.63)	0.52 (0.44–0.63)	**0.47 (0.38–0.56**)	0.57 (0.5–0.69)	0.57 (0.5–0.63)	0.58 (0.5–0.63)
Specificity	0.71 (0.63–0.75)	0.6 (0.5–0.69)	**0.78 (0.69–0.88**)	0.63 (0.56–0.7)	**0.72 (0.63–0.81**)	**0.72 (0.63–0.81**)
PPV**^*a*^**	0.66 (0.6–0.73)	**0.57 (0.5–0.63**)	**0.7 (0.62–0.75**)	0.61 (0.54–0.67)	**0.68 (0.62–0.73**)	**0.69 (0.6–0.75**)
NPV**^*a*^**	0.62 (0.57–0.67)	**0.56 (0.5–0.6**)	0.6 (0.55–0.63)	0.6 (0.53–0.65)	0.63 (0.59–0.67)	0.63 (0.57–0.67)

^*a*^ AUC: area under the receiver-operating characteristic curve; PRAUC: area under the precision-recall curve; PPV: positive predictive value; NPV: negative predictive value.

^*b*^ Median values and interquartile range are shown. Bold values are significantly different (*P* < 0.05) from ASVs alone by Tukey multiple pairwise-comparison *P* value following one-way ANOVA. Tukey multiple pairwise-comparison *P* value following one-way ANOVA for all comparisons can be found in Table S6.

Use of *K. pneumoniae* genotype as the sole input variables led to a median model performance that was greater than that of clinical factors alone (Fig. 4A). This finding is expected, as 27 genes used as input variables are known to be associated with cases in our previous study (18), whereas most clinical factors were not associated with case status in our original cohort study (7). Interestingly, addition of ASVs to *K. pneumoniae* genotype enhanced model performance (Fig. 4A). Integration of all three data sets led to the highest median model performance, though performance was similar to models using only ASVs and *K. pneumoniae* genotype (Fig. 4A). Other model performance metrics yielded comparable results (Table 3), and use of OTUs with *K. pneumoniae genotype* subtly but significantly diminished performance compared to ASVs (Table S4). Importantly, our highest-performing model significantly outperformed a model where case and control status were randomized (Fig. 4B), and as was observed in Fig. 2D, omission of all ASV000001 and ASV000019 did not impact model performance (Fig. 4C).

Finally, we limited the input data to differential ASVs (Fig. 3F) and *K. pneumoniae* genotype. Model performance using limited ASVs was similar to that of inclusion of all ASVs (“Minimal ASVs,” Fig. 4D). Then, we limited *K. pneumoniae* genotype to the five genes we validated in our previous study using a geographically independent cohort of *K. pneumoniae*-colonized patients (18). Model performance using both the limited ASV and genotype sets also led to slightly reduced model performance compared to the model built on the complete data sets (“Minimal ASVs and Minimal Genes,” Fig. 4D). Both models performed better than a random model. In total, these data indicate that the inclusion of ASVs with *K. pneumoniae* genotype leads to peak model performance. This suggests that the gut community profile of patients colonized by *K. pneumoniae* can be combined with other infection-associated variables to discriminate, and potentially predict, infection in these patients with reasonable confidence.

## DISCUSSION

In this study, we have described the gut community of *K. pneumoniae*-colonized patients and demonstrated that the gut community differs between patients who remain asymptomatic (controls) and those who acquire a subsequent symptomatic infection with their colonizing strain (cases). In machine learning models built on this data, *K. pneumoniae* relative abundance had the greatest feature importance, though other gut microbes were also informative. Interestingly, clinical factors such as antibiotic exposure poorly discriminated cases and controls, whereas a combination of gut community data and *K. pneumoniae* genotype classified cases and controls more accurately than a random model (Fig. 4B), ASVs alone, or genotype alone (Fig. 4A). Collectively, this study demonstrates that the gut community of *K. pneumoniae*-colonized patients can be integrated with other biomarkers (patient factors or *K. pneumoniae* genotype) to assess infection risk. Moreover, these results suggest that there is a potentially important role for the gut microbiota community structure in determining the outcome of *K. pneumoniae* colonization.

Many microbiome studies classify individuals at risk for or experiencing disease as being in a state of dysbiosis; however, this imprecise term often lacks the context of the definition of a healthy microbiome. This is critical for establishing a causal link between the gut microbiome and disease, especially as the microbiome gradually shifts with age, environment, diet, healthcare exposure, and yet undiscovered variables [reviewed in reference (24)]. The goal of the present study is not to indicate that the gut microbiome of *K. pneumoniae*-colonized patients is in a state of health or dysbiosis. Rather, the goal is to identify biomarkers that classify infection in colonized patients. Ideally, the observations here will be tested experimentally to explore a causal role in disease. For example, *Akkermansia* (ASV000021) is currently being considered as a probiotic therapy due to its positive impacts on health (25–27). Yet, in this study, *Akkermansia* is important for model performance (Fig. 2C) but is relatively low abundance and not enriched in cases (Fig. 3C and D). This finding highlights differences between machine learning and classic linear discriminant analysis approaches for identifying sequences associated with specific communities. It may be the case that the ASVs identified through linear discriminant analysis have occult interactions with one another and/or other ASVs that explain the differential outcomes of these approaches. For example, recent experimental findings have determined antagonistic interactions exist between *K. pneumoniae* and *Escherichia coli* as a function of microbial diversity (28). Such interactions may be occurring in this study, as OTU00003 *Escherichia/Shigella* and OTU00001 *Klebsiella* were important and differential drivers of different gut community states (Fig. 1C). Similarly, laboratory experiments demonstrate that members of the *K. oxytoca* complex can reduce *K. pneumoniae* gut colonization (15). Here, we observed that the ASV that most likely represents the majority of the *K. pneumoniae* complex (ASV000001) is absent in patients colonized by the ASV that most likely represents the majority of the *K. oxytoca* complex (ASV000019, Fig. S5C). Despite a potential probiotic effect against *K. pneumoniae*, *K. oxytoca* is a pathogen that is often highly antimicrobial resistant [reviewed in reference (22)]. Therefore, while microbial competition with *K. pneumoniae* may explain this finding, characterizing *K. oxytoca* as a member of a healthy or dysbiotic gut microbiome remains in question. Further exploration of the gut community structures identified in this study is necessary to determine their importance in influencing infection risk in *K. pneumoniae*-colonized patients and therein define dysbiosis and its role in infection risk in this patient population.

The variables that are most important in classifying cases and controls likely differ between pathogens and patient populations. For example, clinical biomarkers do not appear to be critical for discriminating case status in this study (Fig. 4A; Fig. S5). This is in contrast to studies performed at the same clinical site leveraging electronic health records to stratify the risk of complicated *Clostridium* (*Clostridioides*) *difficile* infection (29), suggesting a disease-specific effect where the utility of these clinical data in making predictions varies across tasks. Notably, several of the most abundant OTUs described here are consistent with previous cohorts at the same location, indicating some continuity of gut community structure within this geographical space (5). Other studies of *K. pneumoniae*-colonized individuals, including those colonized with multidrug-resistant (MDR) *K. pneumoniae* strains, report varying degrees of differing gut community structures, ranging from somewhat similar (30) to quite different (31, 32) than what is reported here based on most abundant OTUs. It is worth noting that it can be challenging to directly compare such studies due to differences in sample acquisition, data processing methods, and which data are reported. Similarly, the finding that ASVs yield the optimal taxonomical resolution for classifying case status (Fig. 2) is interesting. A recent machine learning study determined that OTUs were the optimal taxonomical level for predicting colorectal cancer (33). The preference for use of ASVs or OTUs in microbiome studies remains contested (34, 35); however, our study supports the premise that optimal taxonomical resolution is highly dependent on the patient population and outcomes of interest and does not necessarily favor OTUs or ASVs. Moreover, this study indicates that ASVs are not appropriately sensitive for strain-level data resolution for *K. pneumoniae*. This finding supports the growing body of research that culture-based approaches remain the gold standard for strain- and clone-level interrogation of gut microbial community structure (36). Ideally, clinical studies interrogating the role of the microbiome in disease would report both OTU and ASV data when using 16S rRNA gene sequencing. This would allow a better understanding of the role of taxonomical resolution in patient-based studies.

An important facet of this study population compared to other study populations is the diversity of colonizing *K. pneumoniae* strains. Often, studies aimed at describing the gut microbiota of *K. pneumoniae*-colonized patients capture patients colonized with highly clonal MDR *K. pneumoniae* strains (30, 37). In contrast, >100 unique sequence types of *K. pneumoniae* were identified in this study population, predominantly from non-MDR lineages (7). This may explain the lack of discriminatory power of antibiotic exposure for classifying cases and controls (Fig. S6). Antibiotic exposure may be a more discriminatory variable in cohorts of patients predominantly colonized by MDR strains. The attention given to MDR lineages is of course warranted; however, the majority of *K. pneumoniae* infections are caused by non-MDR lineages (38) and studies have demonstrated that the bulk of colonizing *K. pneumoniae* strains are diverse (39). Genetic differences in *K. pneumoniae* lineage may dictate interactions with gut microbiota that influence infection risk in patients colonized by MDR or hypervirulent *K. pneumoniae*. For example, we identified a *K. pneumoniae* factor canonically associated with hypervirulence, the *ter* operon, as a microbiome-dependent gut fitness factor (11). This locus was associated with infection in a hospital-wide patient cohort (40) but not in this cohort of intensive care and hematology/oncology patients (7). Alternatively, it may be that associations between *K. pneumoniae* and gut microbial community structure influence infection risk in a conserved manner. This would be ideal, as it would potentiate novel means for determining infection risk. This highlights the importance of studying all lineages with pathogenic potential to enable accurate risk assessment in colonized patients to reduce the burden of *K. pneumoniae* disease.

Though this study adds to our understanding of the gut microbiome of *K. pneumoniae*-colonized patients, it is not without its limitations. First, we used a case-control design for this study to carefully control for the influence of known and unknown patient factors. However, this study design leads to an overrepresentation of infection in the study population and the modeling metrics should be interpreted only in the context of this study, since in the general population we would expect a much lower infection risk, such as the 4.3% attack rate in our large cohort study from which this nested case-control study was derived (7). Ideally, future studies assessing the role of the microbiome as a risk factor for *K. pneumoniae* infection will accurately represent the true attack rate while capturing a large enough number of patients, both colonized by *K. pneumoniae* and not, to maintain suitable study power. Additionally, there may be uncaptured data, such as antibiotic use or post-discharge adverse healthcare events, that occurred between the rectal swab collection and infection, as the duration between swab collection and infection ranged from 0 to 90 days (7). A comprehensive prospective cohort study that includes regular follow-up is necessary to test the ability of the models presented here to predict infection in colonized patients. Therefore, hypotheses generated in small- and medium-sized studies can be rigorously tested in a study population that reflects the general population. Second, this study is limited in its ability to make functional conclusions about the microbiome due to the use of 16S rRNA gene sequencing instead of metagenomics or other -omics approaches. Unfortunately, many -omics approaches remain cost-restrictive and lack easily testable hypotheses. This and similar studies will aid in the generation of hypotheses that can be tested using these approaches in the future. Third, some patients did not have detectable *Klebsiella* 16S DNA despite microbiological confirmation of colonization. It is possible that our sequencing effort was not deep enough, or DNA extraction was not efficient enough, to capture low-abundance *K. pneumoniae* events, whereas microbiological detection is more sensitive due to our use of selective and differential MacConkey agar. Finally, the use of machine learning models in this study is a useful means of determining the discriminatory ability of a large set of variables but is limited in its interpretability. Clinically actionable risk stratification models should be comprised of a small set of easily observable variables. In our previous studies, we developed practical tools for identifying biomarkers in *K. pneumoniae*-colonized patients including measurement of *K. pneumoniae* relative abundance and detection of infection-associated genes by PCR (7, 8). We hope that additional practical tools to assess the role of the microbiome in infection risk in *K. pneumoniae*-colonized patients will be developed and integrated with our previously developed tools.

The addition of this study to our collection of studies assessing patient factors, gut dominance, and *K. pneumoniae* genotype (7, 8, 18) represents one of the most comprehensive explorations of infection risk in a cohort of *K. pneumoniae*-colonized patients. Ultimately, this study provides a foundational framework for the development of integrated, actionable models for predicting and stratifying infection risk in *K. pneumoniae-*colonized patients.

## MATERIALS AND METHODS

### Study subject selection

Subjects in the present study were selected based on matching criteria, the availability of rectal swab DNA (8), and whole-genome sequencing data corresponding to the colonizing *K. pneumoniae*, *K. variicola*, or *K. quasipneumoniae* strain (18).

### 16S rRNA gene sequencing and data processing

DNA was previously extracted from patient rectal swabs (8) using the MagAttract PowerMicrobiome DNA/RNA Kit (Qiagen) and an epMotion 5075 liquid handling system. Standard PCRs used 1, 2, or 7 µL of undiluted DNA and touchdown PCR used 7 µL of undiluted DNA to amplify the V4 region of the 16S rRNA gene. Sequencing was performed as previously described (41). 16S rRNA gene sequences were processed with mothur (v.1.48.0) (19, 42). The sequencing error rate was assessed using a predefined mock community and estimated to be 0.033%. Sequences were aligned to the SILVA reference alignment, release 132 (43) and binned into OTUs using the OptiClust method (44) based on 97% sequence similarity or kept as unique sequences for ASVs. Taxonomic composition was assigned by classifying sequences within mothur using a modified version of the Ribosomal Database Project training set, version 18 (45, 46). Data processing was performed using the Great Lakes High-Performance Computing Cluster at the University of Michigan, Ann Arbor or the Carbonate large-memory computer cluster at Indiana University.

### Data analysis

Data analysis was carried out in RStudio 2021.09.0+351 “Ghost Orchid” Release for macOS or in R, v.4.2.0. R was used instead of RStudio when the analysis was being performed on The Great Lakes High-Performance Computing Cluster at the University of Michigan, Ann Arbor or the Carbonate large-memory computer cluster at Indiana University. For all analyses, sample read counts were rarefied to the lowest-abundance sample (4,438 reads). Alpha- and beta-diversity, principal coordinates analysis, and community typing were performed using mothur. *θ*_YC_ was used as the distance metric for principal coordinates analysis. Differences in community structure were assessed by permutational multivariate analysis of variance (PERMANOVA, 1,000 permutations) from the vegan package, v.2.6-2 (47). Differences in alpha-diversity indices were assessed by Student’s *t* test using the stats package, v.3.6.2. Assessment of differentially enriched OTUs and ASVs was performed with linear discriminant analysis effect size analysis. Supervised machine learning was performed using mikropml, v.1.4.0 (48). First, continuous data were split into quartiles, then input data were preprocessed in mikropml using the default settings. All cases (*N* = 83) and 83 controls were randomly selected for each model iteration. Supervised machine learning was performed using case status as the outcome. Input data were split 80:20 into train and test groups. An optimal model was trained using 100× 5-fold cross-validation and model performance was evaluated using the test data. For regularized logistic regression, hyperparameter selection was semi-automated. Each model was trained with alpha values ranging from 0 to 1, iterated in steps of 0.1, permutated with lambda values ranging from 10^−4^ to 10^1^, iterated in steps of 3 between each log (e.g., 10^−4^, 2.5 × 10^−4^, 5 × 10^−4^, 7.5 × 10^–4^, 10^−3^, 2.5 × 10^−3^…10^1^). Hyperparameters that yielded the best performance were selected to evaluate model performance using the test data. This process was parallelized 100 times, using 100 different seeds to determine the train:test data split, and feature importance and weight was determined for all variables. Model performance metrics were defined as follows: Area under the receiver-operating characteristic curve (AUC), which is a calculation of the area under curve generated by plotting the true positive rate as a function of the false positive rate, wherein a value 0.5 is random classification of outcomes and 1.0 is perfect classification of outcomes; Area under the precision-recall curve (PRAUC), which is a calculation of the area under a curve generated by plotting precision (also known as positive predictive value) as a function of recall (also known as sensitivity), wherein a value 0.5 is random classification of outcomes and 1.0 is perfect classification of outcomes; $Sensitivity=\frac{True\;positives}{True\;positives+False\;negatives};Specificity=\frac{True\;negatives}{True\;negative+False\;positives};$$Positive\;predictive\;value\;\left(PPV\right)=\frac{True\;positives}{True\;positives+False\;positives};Negative\;predictive\;value\;\left(NPV\right)=\frac{True\;negatives}{True\;negatives+False\;negatives}.$

Network analysis was performed using NetCoMi v.1.1.0 (49). Networks were constructed using the compositionally aware correlation estimators, SparCC (50), and networks were compared by permutation test with 100 permutations. For all analyses, a *P* value ≤ 0.05 after Benjamini-Hochberg adjustment was considered statistically significant. Data were visualized using ggplot2, v.4.1.2 (51).

## Supplementary Material

Reviewer comments

## Data Availability

The sequencing data generated in this study have been deposited in the Sequence Read Archive (SRA) database under accession PRJNA789565. Deidentified human data are available under restricted access and can be obtained from M.A.B. within 1 year upon request, pending approval from the University of Michigan Institutional Review Board. All other source data and code are available at https://github.com/jayvorn/Gut-community-structure-as-a-risk-factor-for-infection-in-Klebsiella-colonized-patients.

## SUPPLEMENTAL MATERIAL

Supplemental figures and tables msystems.00786-24-s0001.docx Fig. S1-S6 and Tables S1-S6.

OPEN PEER REVIEW reviewer-comments.pdf An accounting of the reviewer comments and feedback.

ASM does not own the copyrights to Supplemental Material that may be linked to, or accessed through, an article. The authors have granted ASM a non-exclusive, world-wide license to publish the Supplemental Material files. Please contact the corresponding author directly for reuse.
