# Supplementary material for: Gut community structure as a risk factor for infection in Klebsiella pneumoniae-colonized patients
Source: mSystems. 2024 Jul 8;9(8):e00786-24. doi: 10.1128/msystems.00786-24 (PMC11334466; doi:10.1128/msystems.00786-24)
Supplement: Supplemental figures and tables — Fig. S1-S6 and Tables S1-S6. [file msystems.00786-24-s0001.docx]

Supplementary Information


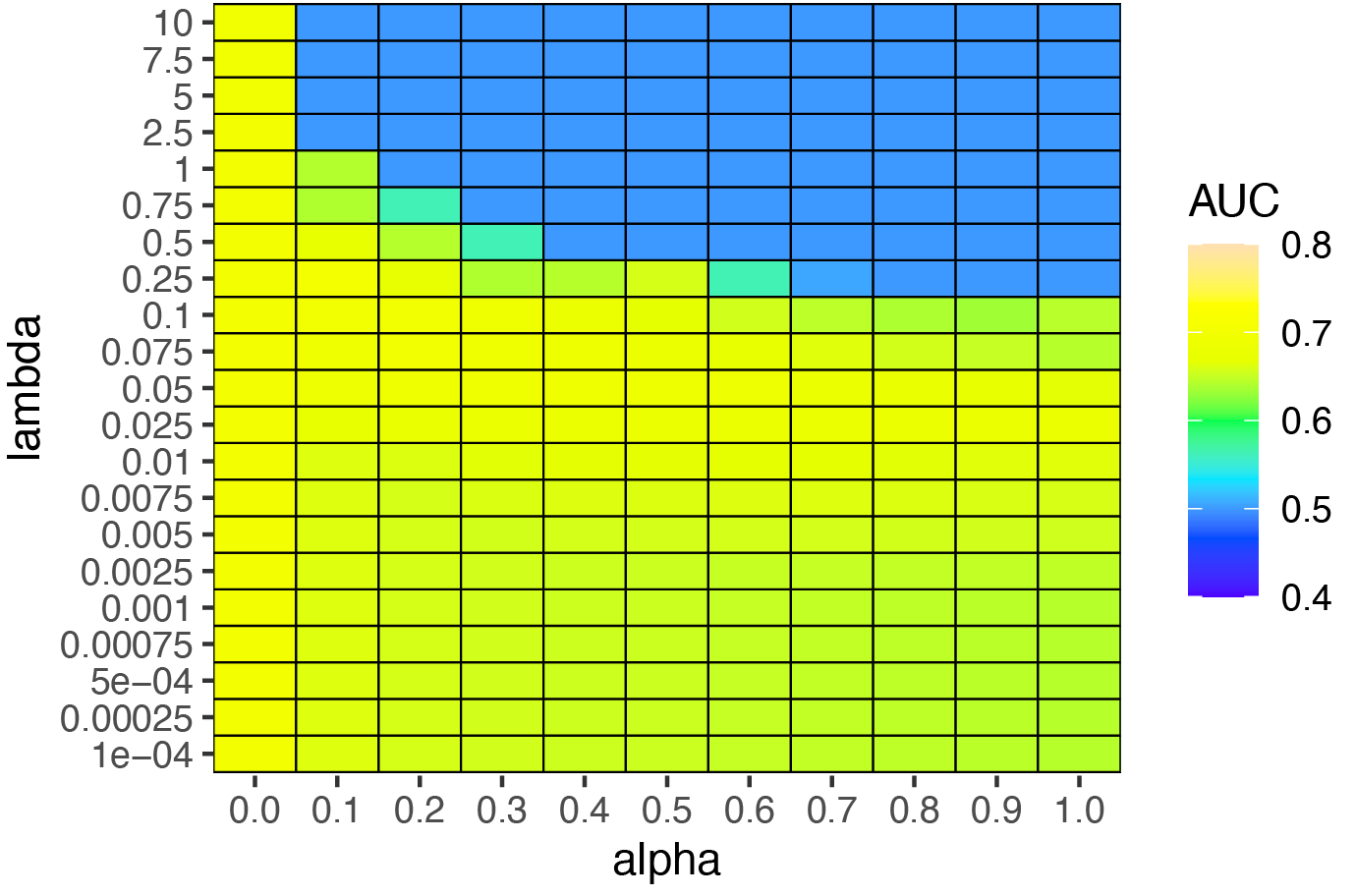


Figure S1. Example of regularized logistic regression hyperparameter selection.

Regularized logistic regression trained model performance as measured by area under the receiver-operator characteristic curve (AUC) for 100 seeds. ASVs were used as input data. Each box contains the mean AUC values for each hyperparameter combination. Future model testing was performed using the hyperparameter combination that yielded the peak AUC for a given seed.


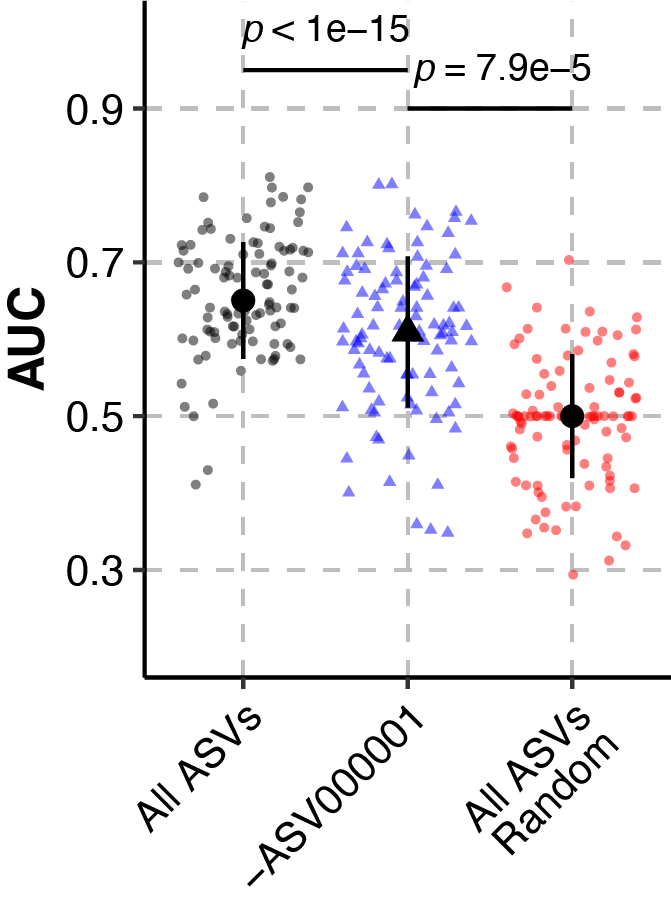


Figure S2. Exclusion of ASV000001 reduces model performance but does not lead to an uninformative model.

Regularized logistic regression model performance on test data sets for 100 seeds predicting case status in *Klebsiella* colonized patients using all ASVs (All ASVs) or excluding ASV000001. Black circles indicate median values, black lines indicate standard deviation, and *p* indicates Tukey multiple pairwise-comparison *p-*value following one way ANOVA. Each datapoint indicates one test data set.


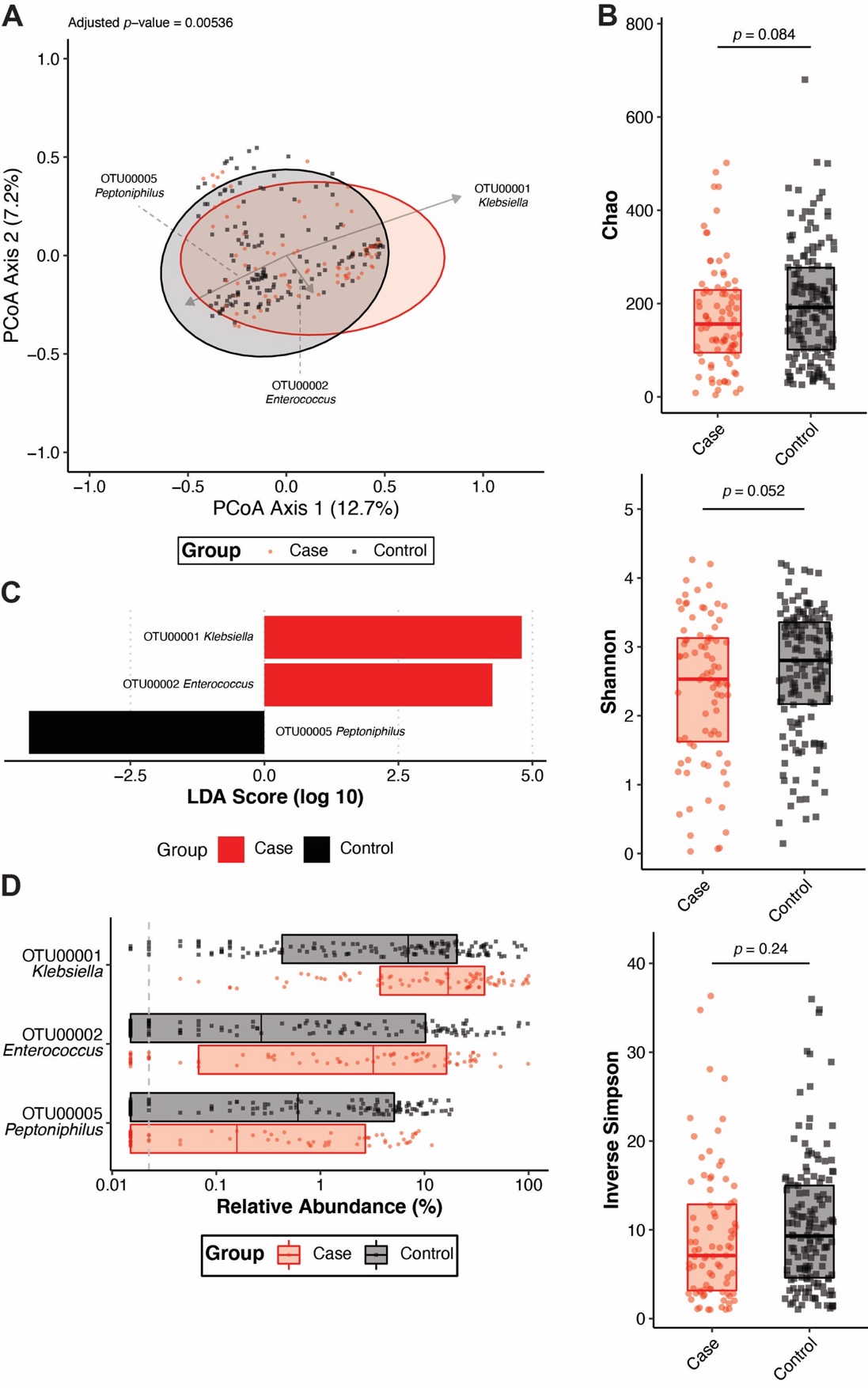


Figure S3. Cases and controls have distinct gut community profiles based on OTUs

(A) Principal coordinates analysis with overlayed biplots of specific OTUs. Analysis of molecular variance (AMOVA) based on the Yue and Clayton θ dissimilarity index was used to assess the difference in beta-diversity between cases (N = 83) and controls (N = 149). (B) Analysis of the Chao, Shannon, and Inverse Simpson alpha-diversity indices between cases (N = 83) and controls (N = 149, boxplot indicates median with interquartile range, *p* indicates student’s *t* test *p-*value). (C) Linear discriminant analysis (LDA) effect size was used to identify differentially abundant OTUs between cases (N = 83) and controls (N = 149). (D) Summary of relative abundances of OTUs that were differentially abundant between cases (N = 83) and controls (N = 149, boxplot indicates median with interquartile range). For all panels, each datapoint indicates one patient.


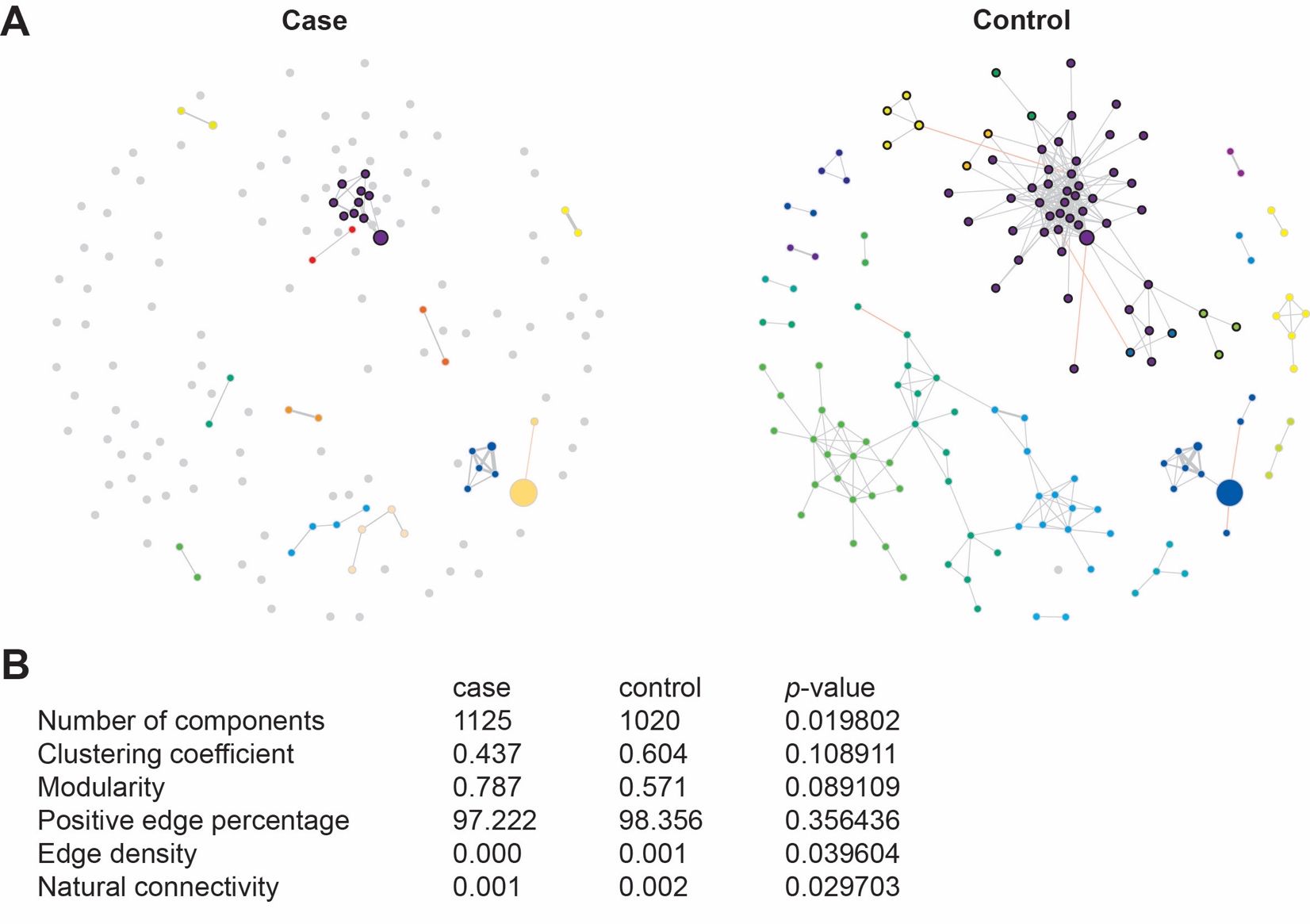


Figure S4. Cases and controls have distinct gut community networks

(A) Network plots of case and control gut communities. Only ASVs with ≥1,000 total reads were included in network construction, and only nodes with significant correlations (student’s *t*-test Benjamini & Hochberg corrected *p-*value < 0.05) in either group are shown. Each node is a single ASV, scaled to the total read count. Grey edges are positive correlations, and orange edges are negative correlations. Node colors indicate distinct clusters. (B) Permutation test (N = 100 permutations) results comparing case and control network properties.


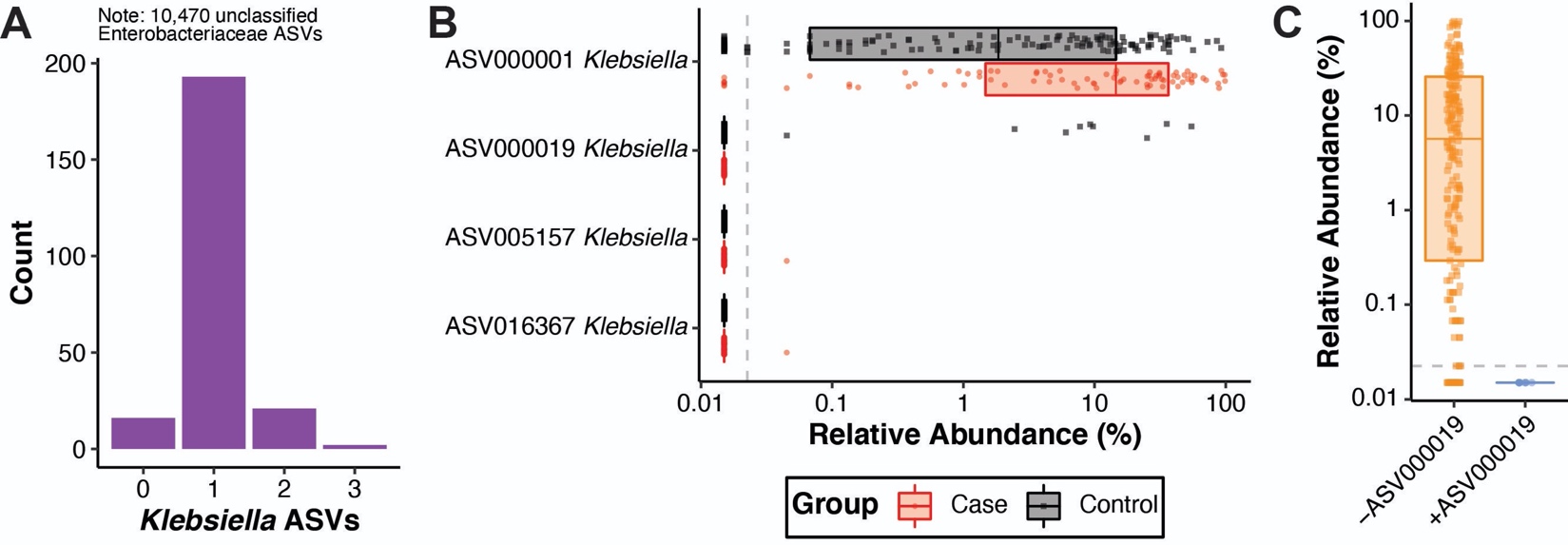


Figure S5. Multiple *Klebsiella* ASVs are present in *Klebsiella*-colonized patients

(A) Count of the number of *Klebsiella* ASVs in each patient (N = 232). (B) Summary of relative abundances of *Klebsiella* ASVs with a sequence count >1 stratified by cases (N = 83) and controls (N = 149, boxplot indicates median with interquartile range). (C) Summary of relative abundance of ASV000001 when ASV000019 is absent (-ASV000019, N = 223) or present (+ASV000019, N = 9, boxplot indicates median with interquartile range). For panels B and C, each datapoint indicates one patient.


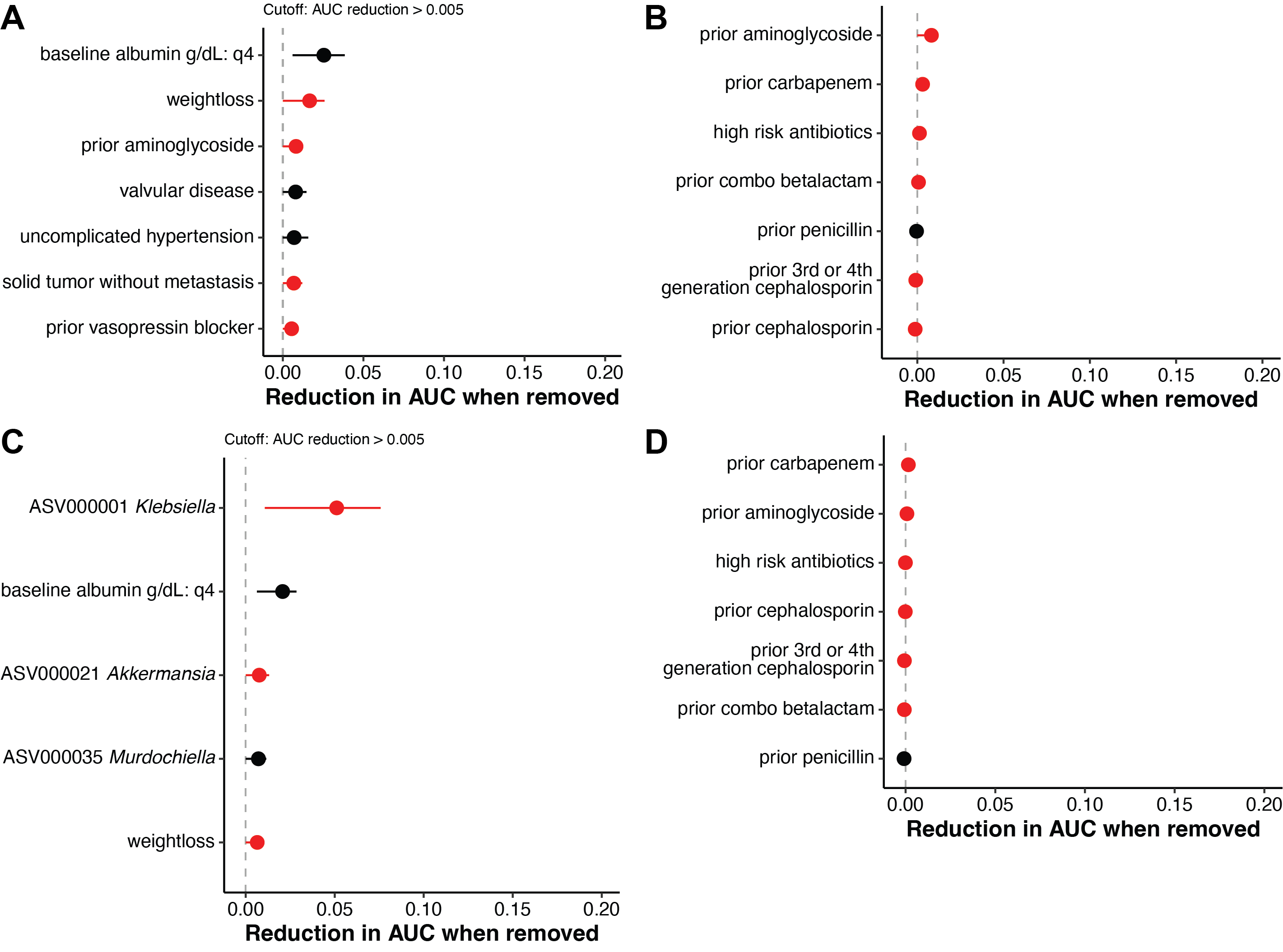


Figure S6. Antibiotic exposure is not important for classifying cases and controls

(A) Top model features for regularized logistic regression models using clinical variables as input data, corresponding to Figure 4 “clinical.” (B) Antibiotic exposure features for regularized logistic regression models using clinical variables as input data, corresponding to Figure 4 “clinical.” (C) Top model features for regularized logistic regression models using clinical variables and ASVs as input data, corresponding to Figure 4 “ASV+clinical.” (D) Antibiotic exposure features for regularized logistic regression models using clinical variables as input data, corresponding to Figure 4 “ASV+clinical.” For all panels, circles indicate mean feature importance and lines indicate interquartile range. Feature importance values in red and black indicate a regression weight that are weighted toward cases and controls, respectively.

Table S1. Sample partitions

| sample | case status | 2 partition | 3 partition |
| --- | --- | --- | --- |
| PR14363 | control | partition 2 | partition 1 |
| PR14545 | control | partition 2 | partition 2 |
| PR14583 | case | partition 1 | partition 3 |
| PR14615 | control | partition 2 | partition 1 |
| PR14638 | control | partition 2 | partition 2 |
| PR15215 | control | partition 1 | partition 3 |
| PR15313 | case | partition 1 | partition 3 |
| PR15410 | case | partition 1 | partition 3 |
| PR15430 | case | partition 2 | partition 2 |
| PR15560 | control | partition 1 | partition 3 |
| PR15562 | control | partition 1 | partition 3 |
| PR15788 | case | partition 1 | partition 3 |
| PR15904 | case | partition 1 | partition 3 |
| PR16045 | control | partition 2 | partition 2 |
| PR16059 | control | partition 1 | partition 3 |
| PR16254 | control | partition 2 | partition 2 |
| PR16263 | case | partition 2 | partition 2 |
| PR16266 | control | partition 2 | partition 2 |
| PR16461 | control | partition 2 | partition 2 |
| PR16503 | control | partition 1 | partition 3 |
| PR16633 | control | partition 2 | partition 2 |
| PR16709 | case | partition 2 | partition 1 |
| PR16746 | case | partition 2 | partition 2 |
| PR16770 | case | partition 2 | partition 2 |
| PR16832 | control | partition 2 | partition 2 |
| PR16969 | control | partition 2 | partition 1 |
| PR17000 | control | partition 2 | partition 2 |
| PR17044 | control | partition 2 | partition 2 |
| PR17117 | control | partition 1 | partition 3 |
| PR17168 | control | partition 1 | partition 3 |
| PR17194 | case | partition 2 | partition 2 |
| PR17318 | control | partition 2 | partition 2 |
| PR17332 | control | partition 2 | partition 2 |
| PR17429 | case | partition 2 | partition 2 |
| PR17433 | control | partition 2 | partition 2 |
| PR17482 | control | partition 1 | partition 3 |
| PR17584 | control | partition 1 | partition 3 |
| PR17665 | control | partition 2 | partition 1 |
| PR17699 | control | partition 2 | partition 2 |
| PR17703 | case | partition 2 | partition 2 |
| PR17713 | control | partition 2 | partition 1 |
| PR17781 | control | partition 1 | partition 3 |
| PR17790 | control | partition 1 | partition 3 |
| PR17853 | control | partition 2 | partition 2 |
| PR17929 | case | partition 2 | partition 1 |
| PR17967 | control | partition 1 | partition 3 |
| PR18060 | case | partition 1 | partition 3 |
| PR18088 | case | partition 1 | partition 2 |
| PR18104 | control | partition 1 | partition 3 |
| PR18133 | case | partition 1 | partition 3 |
| PR18166 | case | partition 1 | partition 2 |
| PR18170 | case | partition 2 | partition 2 |
| PR18174 | control | partition 2 | partition 2 |
| PR18203 | control | partition 1 | partition 3 |
| PR18215 | control | partition 2 | partition 2 |
| PR18227 | control | partition 1 | partition 3 |
| PR18291 | case | partition 2 | partition 2 |
| PR18332 | control | partition 2 | partition 2 |
| PR18358 | control | partition 2 | partition 2 |
| PR18399 | case | partition 2 | partition 2 |
| PR18510 | case | partition 1 | partition 3 |
| PR18520 | control | partition 2 | partition 2 |
| PR18599 | case | partition 1 | partition 3 |
| PR18603 | control | partition 2 | partition 1 |
| PR18638 | control | partition 1 | partition 3 |
| PR18797 | control | partition 2 | partition 2 |
| PR18801 | case | partition 1 | partition 3 |
| PR18838 | control | partition 2 | partition 2 |
| PR18891 | control | partition 2 | partition 1 |
| PR18900 | case | partition 1 | partition 3 |
| PR18922 | case | partition 1 | partition 3 |
| PR18975 | control | partition 1 | partition 1 |
| PR19028 | control | partition 2 | partition 2 |
| PR19076 | control | partition 2 | partition 2 |
| PR19438 | control | partition 2 | partition 2 |
| PR19612 | case | partition 1 | partition 3 |
| PR19821 | control | partition 2 | partition 2 |
| PR19881 | control | partition 1 | partition 3 |
| PR20124 | control | partition 2 | partition 2 |
| PR20197 | control | partition 2 | partition 1 |
| PR20293 | control | partition 1 | partition 2 |
| PR20569 | control | partition 2 | partition 2 |
| PR20588 | control | partition 2 | partition 2 |
| PR20610 | control | partition 2 | partition 2 |
| PR20649 | control | partition 2 | partition 2 |
| PR20670 | case | partition 2 | partition 2 |
| PR20723 | case | partition 2 | partition 1 |
| PR20815 | case | partition 2 | partition 2 |
| PR20827 | control | partition 2 | partition 1 |
| PR20860 | case | partition 1 | partition 3 |
| PR20876 | case | partition 1 | partition 3 |
| PR21010 | control | partition 2 | partition 2 |
| PR21255 | control | partition 2 | partition 2 |
| PR21276 | control | partition 1 | partition 3 |
| PR21324 | case | partition 2 | partition 2 |
| PR21373 | case | partition 2 | partition 2 |
| PR21593 | control | partition 2 | partition 2 |
| PR21604 | control | partition 1 | partition 3 |
| PR21624 | control | partition 1 | partition 3 |
| PR21703 | case | partition 1 | partition 3 |
| PR21718 | case | partition 1 | partition 3 |
| PR21884 | control | partition 1 | partition 3 |
| PR21917 | control | partition 2 | partition 2 |
| PR21959 | case | partition 2 | partition 2 |
| PR22037 | control | partition 2 | partition 1 |
| PR22162 | control | partition 2 | partition 2 |
| PR22265 | case | partition 1 | partition 3 |
| PR22356 | case | partition 1 | partition 3 |
| PR22440 | control | partition 1 | partition 3 |
| PR22540 | control | partition 1 | partition 3 |
| PR22591 | case | partition 1 | partition 3 |
| PR22592 | control | partition 1 | partition 1 |
| PR22826 | control | partition 1 | partition 3 |
| PR22903 | control | partition 1 | partition 3 |
| PR23003 | case | partition 2 | partition 1 |
| PR23187 | control | partition 1 | partition 3 |
| PR23271 | control | partition 1 | partition 2 |
| PR23568 | case | partition 2 | partition 2 |
| PR24010 | control | partition 2 | partition 2 |
| PR24311 | control | partition 1 | partition 3 |
| PR24548 | control | partition 1 | partition 3 |
| PR24563 | control | partition 1 | partition 3 |
| PR24588 | control | partition 2 | partition 2 |
| PR24687 | control | partition 2 | partition 2 |
| PR24765 | control | partition 1 | partition 3 |
| PR24778 | case | partition 2 | partition 2 |
| PR24803 | control | partition 2 | partition 2 |
| PR24863 | case | partition 2 | partition 2 |
| PR24930 | control | partition 1 | partition 1 |
| PR25045 | control | partition 1 | partition 3 |
| PR25064 | control | partition 2 | partition 2 |
| PR25082 | case | partition 2 | partition 2 |
| PR25083 | control | partition 2 | partition 2 |
| PR25100 | control | partition 2 | partition 2 |
| PR25160 | case | partition 2 | partition 2 |
| PR25239 | case | partition 2 | partition 1 |
| PR25320 | control | partition 2 | partition 2 |
| PR25343 | control | partition 2 | partition 2 |
| PR25413 | case | partition 1 | partition 3 |
| PR25434 | case | partition 2 | partition 2 |
| PR25486 | control | partition 1 | partition 3 |
| PR25565 | case | partition 2 | partition 2 |
| PR25581 | control | partition 1 | partition 3 |
| PR25587 | control | partition 2 | partition 1 |
| PR25723 | case | partition 1 | partition 3 |
| PR25832 | case | partition 2 | partition 2 |
| PR25883 | control | partition 1 | partition 3 |
| PR25897 | case | partition 1 | partition 3 |
| PR25909 | case | partition 1 | partition 1 |
| PR25957 | case | partition 1 | partition 3 |
| PR26017 | control | partition 1 | partition 3 |
| PR26083 | control | partition 1 | partition 3 |
| PR26327 | case | partition 2 | partition 2 |
| PR26333 | case | partition 2 | partition 2 |
| PR26410 | control | partition 1 | partition 3 |
| PR26421 | control | partition 1 | partition 3 |
| PR26485 | control | partition 2 | partition 2 |
| PR26491 | control | partition 2 | partition 2 |
| PR26498 | control | partition 1 | partition 3 |
| PR26564 | control | partition 1 | partition 3 |
| PR26666 | control | partition 2 | partition 2 |
| PR26691 | control | partition 1 | partition 1 |
| PR26715 | control | partition 2 | partition 2 |
| PR26774 | control | partition 1 | partition 3 |
| PR26893 | control | partition 1 | partition 3 |
| PR26959 | case | partition 1 | partition 2 |
| PR27029 | control | partition 2 | partition 2 |
| PR27184 | case | partition 2 | partition 2 |
| PR27193 | case | partition 2 | partition 1 |
| PR27201 | case | partition 1 | partition 3 |
| PR27221 | control | partition 2 | partition 2 |
| PR27307 | control | partition 1 | partition 3 |
| PR27309 | control | partition 1 | partition 3 |
| PR27323 | control | partition 1 | partition 3 |
| PR27351 | case | partition 2 | partition 2 |
| PR27841 | control | partition 2 | partition 1 |
| PR27885 | control | partition 2 | partition 2 |
| PR27893 | control | partition 2 | partition 2 |
| PR28009 | control | partition 2 | partition 2 |
| PR28069 | control | partition 2 | partition 2 |
| PR28122 | control | partition 1 | partition 2 |
| PR28135 | control | partition 1 | partition 3 |
| PR28152 | control | partition 2 | partition 2 |
| PR28241 | case | partition 1 | partition 3 |
| PR28314 | control | partition 1 | partition 3 |
| PR28337 | control | partition 2 | partition 2 |
| PR28635 | control | partition 2 | partition 1 |
| PR28636 | control | partition 2 | partition 2 |
| PR28720 | case | partition 2 | partition 2 |
| PR28783 | control | partition 2 | partition 2 |
| PR28861 | control | partition 1 | partition 3 |
| PR28869 | case | partition 1 | partition 3 |
| PR28885 | case | partition 1 | partition 3 |
| PR28912 | control | partition 2 | partition 2 |
| PR28930 | case | partition 2 | partition 2 |
| PR28940 | control | partition 2 | partition 2 |
| PR28990 | control | partition 2 | partition 2 |
| PR29054 | control | partition 1 | partition 3 |
| PR29065 | case | partition 1 | partition 3 |
| PR29077 | control | partition 1 | partition 3 |
| PR29080 | case | partition 1 | partition 3 |
| PR29081 | case | partition 2 | partition 1 |
| PR29089 | control | partition 1 | partition 3 |
| PR29187 | control | partition 1 | partition 1 |
| PR29189 | control | partition 2 | partition 2 |
| PR29272 | case | partition 2 | partition 1 |
| PR29281 | case | partition 1 | partition 3 |
| PR29292 | control | partition 2 | partition 2 |
| PR29346 | control | partition 2 | partition 1 |
| PR29361 | control | partition 2 | partition 1 |
| PR29415 | control | partition 1 | partition 3 |
| PR29420 | control | partition 1 | partition 3 |
| PR29510 | control | partition 2 | partition 1 |
| PR29556 | control | partition 2 | partition 2 |
| PR29656 | case | partition 1 | partition 3 |
| PR29663 | control | partition 2 | partition 2 |
| PR29669 | case | partition 1 | partition 3 |
| PR29826 | control | partition 1 | partition 3 |
| PR29848 | case | partition 1 | partition 2 |
| PR30067 | control | partition 1 | partition 1 |
| PR30391 | control | partition 2 | partition 1 |
| PR30422 | control | partition 1 | partition 3 |
| PR30692 | control | partition 1 | partition 3 |
| PR30710 | control | partition 1 | partition 3 |
| PR30711 | case | partition 1 | partition 3 |
| PR30890 | case | partition 1 | partition 1 |
| PR30974 | case | partition 1 | partition 3 |
| PR31280 | case | partition 2 | partition 1 |
| PR31292 | case | partition 1 | partition 2 |
| PR31436 | case | partition 1 | partition 1 |
| PR31620 | case | partition 2 | partition 1 |
| PR31764 | case | partition 2 | partition 2 |

Table S2. BLAST alignment* to ASV000001 and ASV000019 rRNA sequences.

| Query | Subject | Species complex | Accession |
| --- | --- | --- | --- |
| ASV000001 | *Klebsiella pneumoniae* | *K. pneumoniae* | NR 036794.1 |
| ASV000001 | *Klebsiella pneumoniae* | *K. pneumoniae* | NR 112009.1 |
| ASV000001 | *Klebsiella pneumoniae* | *K. pneumoniae* | NR 113240.1 |
| ASV000001 | *Klebsiella pneumoniae* | *K. pneumoniae* | NR 113702.1 |
| ASV000001 | *Klebsiella pneumoniae* | *K. pneumoniae* | NR 114506.1 |
| ASV000001 | *Klebsiella pneumoniae* | *K. pneumoniae* | NR 114715.1 |
| ASV000001 | *Klebsiella pneumoniae* | *K. pneumoniae* | NR 117682.1 |
| ASV000001 | *Klebsiella pneumoniae* | *K. pneumoniae* | NR 117683.1 |
| ASV000001 | *Klebsiella pneumoniae* | *K. pneumoniae* | NR 117684.1 |
| ASV000001 | *Klebsiella pneumoniae* | *K. pneumoniae* | NR 117685.1 |
| ASV000001 | *Klebsiella pneumoniae* | *K. pneumoniae* | NR 117686.1 |
| ASV000001 | *Klebsiella pneumoniae* | *K. pneumoniae* | NR 119278.1 |
| ASV000001 | *Klebsiella pneumoniae* subsp. *rhinoscleromatis* | *K. pneumoniae* | NR 037084.1 |
| ASV000001 | *Klebsiella pneumoniae* subsp. *rhinoscleromatis* ATCC 13884 | *K. pneumoniae* | NR 114507.1 |
| ASV000001 | *Klebsiella quasipneumoniae* subsp. *quasipneumoniae* | *K. pneumoniae* | NR 134062.1 |
| ASV000001 | *Klebsiella quasipneumoniae* subsp. *similipneumoniae* | *K. pneumoniae* | NR 134063.1 |
| ASV000001 | *Klebsiella variicola* | *K. pneumoniae* | NR 025635.1 |
| ASV000001 | *Klebsiella huaxiensis* | *K. oxytoca* | NR 171417.1 |
| ASV000001 | *Klebsiella aerogenes* | NA | NR 024643.1 |
| ASV000001 | *Klebsiella aerogenes* | NA | NR 113614.1 |
| ASV000001 | *Klebsiella aerogenes* | NA | NR 114737.1 |
| ASV000001 | *Klebsiella aerogenes* | NA | NR 118556.1 |
| ASV000001 | *Klebsiella aerogenes* KCTC 2190 | NA | NR 102493.2 |
| ASV000019 | *Klebsiella grimontii* | *K. oxytoca* | NR 159317.1 |
| ASV000019 | *Klebsiella michiganensis* | *K. oxytoca* | NR 118335.1 |
| ASV000019 | *Klebsiella oxytoca* | *K. oxytoca* | NR 041749.1 |
| ASV000019 | *Klebsiella oxytoca* | *K. oxytoca* | NR 112010.1 |
| ASV000019 | *Klebsiella oxytoca* | *K. oxytoca* | NR 113341.1 |
| ASV000019 | *Klebsiella oxytoca* | *K. oxytoca* | NR 114152.1 |
| ASV000019 | *Klebsiella oxytoca* | *K. oxytoca* | NR 118853.1 |
| ASV000019 | *Klebsiella oxytoca* | *K. oxytoca* | NR 119277.1 |

*Only alignments with 100% identity are shown

Table S3. Clinical variables included in machine learning models*

| Variable |  | Case (N = 83) | Control (N = 147) |
| --- | --- | --- | --- |
| depression | yes | 29 (34.9%) | 39 (26.5%) |
|  | no | 54 (65.1%) | 108 (73.5%) |
|  | missing | 0 (0%) | 0 (0%) |
| prior diuretic | yes | 30 (36.1%) | 35 (23.8%) |
|  | no | 53 (63.9%) | 112 (76.2%) |
|  | missing | 0 (0%) | 0 (0%) |
| prior vitamin D | yes | 18 (21.7%) | 18 (12.2%) |
|  | no | 65 (78.3%) | 129 (87.8%) |
|  | missing | 0 (0%) | 0 (0%) |
| prior vasopressin blocker | yes | 19 (22.9%) | 14 (9.5%) |
|  | no | 64 (77.1%) | 133 (90.5%) |
|  | missing | 0 (0%) | 0 (0%) |
| albumin < 2.5 g/dL | yes | 34 (41%) | 34 (23.1%) |
|  | no | 46 (55.4%) | 106 (72.1%) |
|  | missing | 3 (3.6%) | 7 (4.8%) |
| high risk antibiotics | yes | 30 (36.1%) | 30 (20.4%) |
|  | no | 53 (63.9%) | 117 (79.6%) |
|  | missing | 0 (0%) | 0 (0%) |
| weighted elixhauser | mean ± SD | 22.4 ± 11.5 | 19.3 ± 12.5 |
| alcohol abuse | yes | 5 (6%) | 17 (11.6%) |
|  | no | 78 (94%) | 130 (88.4%) |
|  | missing | 0 (0%) | 0 (0%) |
| blood loss anemia | yes | 18 (21.7%) | 17 (11.6%) |
|  | no | 65 (78.3%) | 130 (88.4%) |
|  | missing | 0 (0%) | 0 (0%) |
| cardiac arrhythmias | yes | 50 (60.2%) | 83 (56.5%) |
|  | no | 33 (39.8%) | 64 (43.5%) |
|  | missing | 0 (0%) | 0 (0%) |
| chronic pulmonary disease | yes | 26 (31.3%) | 46 (31.3%) |
|  | no | 57 (68.7%) | 101 (68.7%) |
|  | missing | 0 (0%) | 0 (0%) |
| coagulopathy | yes | 36 (43.4%) | 53 (36.1%) |
|  | no | 47 (56.6%) | 94 (63.9%) |
|  | missing | 0 (0%) | 0 (0%) |
| congestive heart failure | yes | 28 (33.7%) | 47 (32%) |
|  | no | 55 (66.3%) | 100 (68%) |
|  | missing | 0 (0%) | 0 (0%) |
| deficiency anemia | yes | 11 (13.3%) | 20 (13.6%) |
|  | no | 72 (86.7%) | 127 (86.4%) |
|  | missing | 0 (0%) | 0 (0%) |
| complicated diabetes | yes | 15 (18.1%) | 21 (14.3%) |
|  | no | 68 (81.9%) | 126 (85.7%) |
|  | missing | 0 (0%) | 0 (0%) |
| uncomplicated diabetes | yes | 27 (32.5%) | 36 (24.5%) |
|  | no | 56 (67.5%) | 111 (75.5%) |
|  | missing | 0 (0%) | 0 (0%) |
| drug abuse | yes | 6 (7.2%) | 8 (5.4%) |
|  | no | 77 (92.8%) | 139 (94.6%) |
|  | missing | 0 (0%) | 0 (0%) |
| fluid electrolyte disorders | yes | 57 (68.7%) | 99 (67.3%) |
|  | no | 26 (31.3%) | 48 (32.7%) |
|  | missing | 0 (0%) | 0 (0%) |
| complicated hypertension | yes | 33 (39.8%) | 48 (32.7%) |
|  | no | 50 (60.2%) | 99 (67.3%) |
|  | missing | 0 (0%) | 0 (0%) |
| uncomplicated hypertension | yes | 35 (42.2%) | 86 (58.5%) |
|  | no | 48 (57.8%) | 61 (41.5%) |
|  | missing | 0 (0%) | 0 (0%) |
| hypothyroidism | yes | 15 (18.1%) | 16 (10.9%) |
|  | no | 68 (81.9%) | 131 (89.1%) |
|  | missing | 0 (0%) | 0 (0%) |
| liver disease | yes | 19 (22.9%) | 33 (22.4%) |
|  | no | 64 (77.1%) | 114 (77.6%) |
|  | missing | 0 (0%) | 0 (0%) |
| lymphoma | yes | 8 (9.6%) | 19 (12.9%) |
|  | no | 75 (90.4%) | 128 (87.1%) |
|  | missing | 0 (0%) | 0 (0%) |
| metastatic cancer | yes | 15 (18.1%) | 22 (15%) |
|  | no | 68 (81.9%) | 125 (85%) |
|  | missing | 0 (0%) | 0 (0%) |
| obesity | yes | 26 (31.3%) | 39 (26.5%) |
|  | no | 57 (68.7%) | 108 (73.5%) |
|  | missing | 0 (0%) | 0 (0%) |
| other neurological disorders | yes | 24 (28.9%) | 23 (15.6%) |
|  | no | 59 (71.1%) | 124 (84.4%) |
|  | missing | 0 (0%) | 0 (0%) |
| paralysis | yes | 6 (7.2%) | 4 (2.7%) |
|  | no | 77 (92.8%) | 143 (97.3%) |
|  | missing | 0 (0%) | 0 (0%) |
| peptic ulcer disease excluding bleeding | yes | 5 (6%) | 6 (4.1%) |
|  | no | 78 (94%) | 141 (95.9%) |
|  | missing | 0 (0%) | 0 (0%) |
| peripheral vascular disorders | yes | 17 (20.5%) | 41 (27.9%) |
|  | no | 66 (79.5%) | 106 (72.1%) |
|  | missing | 0 (0%) | 0 (0%) |
| psychoses | yes | 4 (4.8%) | 4 (2.7%) |
|  | no | 79 (95.2%) | 143 (97.3%) |
|  | missing | 0 (0%) | 0 (0%) |
| pulmonary circulation disorders | yes | 14 (16.9%) | 33 (22.4%) |
|  | no | 69 (83.1%) | 114 (77.6%) |
|  | missing | 0 (0%) | 0 (0%) |
| renal failure | yes | 26 (31.3%) | 35 (23.8%) |
|  | no | 57 (68.7%) | 112 (76.2%) |
|  | missing | 0 (0%) | 0 (0%) |
| rheumatoid arthritis collagen vascular diseases | yes | 8 (9.6%) | 10 (6.8%) |
|  | no | 75 (90.4%) | 137 (93.2%) |
|  | missing | 0 (0%) | 0 (0%) |
| solid tumor without metastasis | yes | 26 (31.3%) | 28 (19%) |
|  | no | 57 (68.7%) | 119 (81%) |
|  | missing | 0 (0%) | 0 (0%) |
| valvular disease | yes | 8 (9.6%) | 33 (22.4%) |
|  | no | 75 (90.4%) | 114 (77.6%) |
|  | missing | 0 (0%) | 0 (0%) |
| weight loss | yes | 45 (54.2%) | 49 (33.3%) |
|  | no | 38 (45.8%) | 98 (66.7%) |
|  | missing | 0 (0%) | 0 (0%) |
| urinary catheter | yes | 63 (75.9%) | 88 (59.9%) |
|  | no | 20 (24.1%) | 59 (40.1%) |
|  | missing | 0 (0%) | 0 (0%) |
| feeding tube | yes | 43 (51.8%) | 50 (34%) |
|  | no | 40 (48.2%) | 97 (66%) |
|  | missing | 0 (0%) | 0 (0%) |
| ventilator | yes | 38 (45.8%) | 67 (45.6%) |
|  | no | 45 (54.2%) | 80 (54.4%) |
|  | missing | 0 (0%) | 0 (0%) |
| central line | yes | 54 (65.1%) | 89 (60.5%) |
|  | no | 29 (34.9%) | 58 (39.5%) |
|  | missing | 0 (0%) | 0 (0%) |
| diabetes | yes | 30 (36.1%) | 41 (27.9%) |
|  | no | 53 (63.9%) | 106 (72.1%) |
|  | missing | 0 (0%) | 0 (0%) |
| hypertension | yes | 51 (61.4%) | 100 (68%) |
|  | no | 32 (38.6%) | 47 (32%) |
|  | missing | 0 (0%) | 0 (0%) |
| prior immunosuppressor | yes | 6 (7.2%) | 10 (6.8%) |
|  | no | 77 (92.8%) | 137 (93.2%) |
|  | missing | 0 (0%) | 0 (0%) |
| prior insulin | yes | 24 (28.9%) | 33 (22.4%) |
|  | no | 59 (71.1%) | 114 (77.6%) |
|  | missing | 0 (0%) | 0 (0%) |
| prior hypoglycemics | yes | 0 (0%) | 2 (1.4%) |
|  | no | 83 (100%) | 145 (98.6%) |
|  | missing | 0 (0%) | 0 (0%) |
| prior proton pump inhibitors | yes | 29 (34.9%) | 40 (27.2%) |
|  | no | 54 (65.1%) | 107 (72.8%) |
|  | missing | 0 (0%) | 0 (0%) |
| prior immunoglobulin | yes | 2 (2.4%) | 1 (0.7%) |
|  | no | 81 (97.6%) | 146 (99.3%) |
|  | missing | 0 (0%) | 0 (0%) |
| prior dialysis | yes | 1 (1.2%) | 0 (0%) |
|  | no | 82 (98.8%) | 147 (100%) |
|  | missing | 0 (0%) | 0 (0%) |
| prior nicotine | yes | 1 (1.2%) | 6 (4.1%) |
|  | no | 82 (98.8%) | 141 (95.9%) |
|  | missing | 0 (0%) | 0 (0%) |
| prior angiotensin blocker | yes | 0 (0%) | 8 (5.4%) |
|  | no | 83 (100%) | 139 (94.6%) |
|  | missing | 0 (0%) | 0 (0%) |
| prior antidepressant antipsychotic | yes | 22 (26.5%) | 30 (20.4%) |
|  | no | 61 (73.5%) | 117 (79.6%) |
|  | missing | 0 (0%) | 0 (0%) |
| prior histamine antagonists | yes | 16 (19.3%) | 29 (19.7%) |
|  | no | 67 (80.7%) | 118 (80.3%) |
|  | missing | 0 (0%) | 0 (0%) |
| prior antituberculars | yes | 1 (1.2%) | 1 (0.7%) |
|  | no | 82 (98.8%) | 146 (99.3%) |
|  | missing | 0 (0%) | 0 (0%) |
| prior clindamycin | yes | 3 (3.6%) | 1 (0.7%) |
|  | no | 80 (96.4%) | 146 (99.3%) |
|  | missing | 0 (0%) | 0 (0%) |
| prior cephalosporin | yes | 17 (20.5%) | 20 (13.6%) |
|  | no | 66 (79.5%) | 127 (86.4%) |
|  | missing | 0 (0%) | 0 (0%) |
| prior penicillin | yes | 24 (28.9%) | 26 (17.7%) |
|  | no | 59 (71.1%) | 121 (82.3%) |
|  | missing | 0 (0%) | 0 (0%) |
| prior quinolone | yes | 5 (6%) | 5 (3.4%) |
|  | no | 78 (94%) | 142 (96.6%) |
|  | missing | 0 (0%) | 0 (0%) |
| prior carbapenem | yes | 9 (10.8%) | 3 (2%) |
|  | no | 74 (89.2%) | 144 (98%) |
|  | missing | 0 (0%) | 0 (0%) |
| prior monobactam | yes | 3 (3.6%) | 1 (0.7%) |
|  | no | 80 (96.4%) | 146 (99.3%) |
|  | missing | 0 (0%) | 0 (0%) |
| prior aminoglycoside | yes | 14 (16.9%) | 6 (4.1%) |
|  | no | 69 (83.1%) | 141 (95.9%) |
|  | missing | 0 (0%) | 0 (0%) |
| prior macrolide | yes | 6 (7.2%) | 4 (2.7%) |
|  | no | 77 (92.8%) | 143 (97.3%) |
|  | missing | 0 (0%) | 0 (0%) |
| prior tetracycline | yes | 2 (2.4%) | 3 (2%) |
|  | no | 81 (97.6%) | 144 (98%) |
|  | missing | 0 (0%) | 0 (0%) |
| prior daptomycin | yes | 0 (0%) | 0 (0%) |
|  | no | 83 (100%) | 147 (100%) |
|  | missing | 0 (0%) | 0 (0%) |
| prior rifamycin | yes | 3 (3.6%) | 5 (3.4%) |
|  | no | 80 (96.4%) | 142 (96.6%) |
|  | missing | 0 (0%) | 0 (0%) |
| prior polymyxin | yes | 0 (0%) | 0 (0%) |
|  | no | 83 (100%) | 147 (100%) |
|  | missing | 0 (0%) | 0 (0%) |
| prior fosfomycin | yes | 1 (1.2%) | 0 (0%) |
|  | no | 82 (98.8%) | 147 (100%) |
|  | missing | 0 (0%) | 0 (0%) |
| prior nitrofurantoin | yes | 1 (1.2%) | 2 (1.4%) |
|  | no | 82 (98.8%) | 145 (98.6%) |
|  | missing | 0 (0%) | 0 (0%) |
| prior methotrexate | yes | 0 (0%) | 2 (1.4%) |
|  | no | 83 (100%) | 145 (98.6%) |
|  | missing | 0 (0%) | 0 (0%) |
| prior sulfonamide | yes | 4 (4.8%) | 1 (0.7%) |
|  | no | 79 (95.2%) | 146 (99.3%) |
|  | missing | 0 (0%) | 0 (0%) |
| prior 3rd or 4th generation cephalosporin | yes | 13 (15.7%) | 10 (6.8%) |
|  | no | 70 (84.3%) | 137 (93.2%) |
|  | missing | 0 (0%) | 0 (0%) |
| prior combo betalactam | yes | 24 (28.9%) | 23 (15.6%) |
|  | no | 59 (71.1%) | 124 (84.4%) |
|  | missing | 0 (0%) | 0 (0%) |
| prior linezolid | yes | 4 (4.8%) | 1 (0.7%) |
|  | no | 79 (95.2%) | 146 (99.3%) |
|  | missing | 0 (0%) | 0 (0%) |
| hemoglobin g/dL | mean ± SD | 7.4 ± 1.8 | 8.0 ± 2.1 |
| creatinine mg/dL | mean ± SD | 0.75 ± 0.48 | 0.78 ± 0.50 |
| albumin g/dL | mean ± SD | 2.5 ± 0.71 | 2.8 ± 0.72 |
| protein g/dL | mean ± SD | 4.8 ± 0.95 | 5.0 ± 0.93 |
| new ventilator | yes | 35 (42.2%) | 66 (44.9%) |
|  | no | 48 (57.8%) | 81 (55.1%) |
|  | missing | 0 (0%) | 0 (0%) |
| new urinary catheter | yes | 60 (72.3%) | 84 (57.1%) |
|  | no | 23 (27.7%) | 63 (42.9%) |
|  | missing | 0 (0%) | 0 (0%) |
| new feed tube | yes | 7 (8.4%) | 2 (1.4%) |
|  | no | 76 (91.6%) | 145 (98.6%) |
|  | missing | 0 (0%) | 0 (0%) |
| new central line | yes | 34 (41%) | 69 (46.9%) |
|  | no | 49 (59%) | 78 (53.1%) |
|  | missing | 0 (0%) | 0 (0%) |

*Non-missing data in Table 1 were also included in machine learning models

Table S4. ASV and *K. pneumoniae* genotype versus OTU and *K. pneumoniae* genotype elastic net performance data

|  | ASV + genotype | OTU + genotype |
| --- | --- | --- |
| AUC* | 0.71 (0.66 - 0.76) | **0.68 (0.62 – 0.73)** |
| prAUC* | 0.65 (0.61 - 0.7) | **0.62 (0.58 – 0.69)** |
| Accuracy | 0.65 (0.59 - 0.69) | 0.63 (0.59 – 0.69) |
| Sensitivity | 0.57 (0.5 - 0.63) | 0.56 (0.44 – 0.63) |
| Specificity | 0.72 (0.63 - 0.81) | 0.69 (0.63 – 0.82) |
| PPV* | 0.68 (0.62 - 0.73) | 0.64 (0.59 – 0.72) |
| NPV* | 0.63 (0.59 - 0.67) | 0.61 (0.57 – 0.67) |

Median values and interquartile range are shown. Bold values are significantly different (*p* < 0.05) from ASV + genotype alone by two-sided *t*-test.

*AUC: Area under the receiver-operating characteristic curve; PRAUC: Area under the precision-recall curve; PPV: Positive predictive value; NPV: Negative predictive value

Table S5. Tukey multiple pairwise-comparison *p*-values following one way ANOVA for all taxon level elastic net performance data comparisons.

| Comparison | Metric | Adjusted *p*-value |
| --- | --- | --- |
| Class-ASV | AUC | 0.00E+00 |
| Class-ASV | prAUC | 2.27E-07 |
| Class-ASV | Accuracy | 0.00E+00 |
| Class-ASV | Sensitivity | 9.84E-01 |
| Class-ASV | Specificity | 0.00E+00 |
| Class-ASV | Positive predictive value | 0.00E+00 |
| Class-ASV | Negative predictive value | 0.00E+00 |
| Family-ASV | AUC | 0.00E+00 |
| Family-ASV | prAUC | 0.00E+00 |
| Family-ASV | Accuracy | 0.00E+00 |
| Family-ASV | Sensitivity | 7.02E-01 |
| Family-ASV | Specificity | 0.00E+00 |
| Family-ASV | Positive predictive value | 0.00E+00 |
| Family-ASV | Negative predictive value | 1.61E-08 |
| Family-Class | AUC | 1.00E+00 |
| Family-Class | prAUC | 9.83E-01 |
| Family-Class | Accuracy | 4.14E-01 |
| Family-Class | Sensitivity | 9.89E-01 |
| Family-Class | Specificity | 6.40E-02 |
| Family-Class | Positive predictive value | 4.27E-01 |
| Family-Class | Negative predictive value | 5.78E-01 |
| Genus-ASV | AUC | 1.35E-02 |
| Genus-ASV | prAUC | 7.39E-01 |
| Genus-ASV | Accuracy | 8.48E-04 |
| Genus-ASV | Sensitivity | 9.62E-01 |
| Genus-ASV | Specificity | 3.34E-06 |
| Genus-ASV | Positive predictive value | 1.92E-07 |
| Genus-ASV | Negative predictive value | 1.17E-01 |
| Genus-Class | AUC | 4.26E-06 |
| Genus-Class | prAUC | 4.32E-04 |
| Genus-Class | Accuracy | 3.23E-07 |
| Genus-Class | Sensitivity | 5.67E-01 |
| Genus-Class | Specificity | 5.99E-04 |
| Genus-Class | Positive predictive value | 6.11E-07 |
| Genus-Class | Negative predictive value | 2.63E-06 |
| Genus-Family | AUC | 8.26E-06 |
| Genus-Family | prAUC | 9.16E-06 |
| Genus-Family | Accuracy | 4.01E-03 |
| Genus-Family | Sensitivity | 1.51E-01 |
| Genus-Family | Specificity | 8.37E-01 |
| Genus-Family | Positive predictive value | 6.17E-03 |
| Genus-Family | Negative predictive value | 6.67E-03 |
| Order-ASV | AUC | 0.00E+00 |
| Order-ASV | prAUC | 0.00E+00 |
| Order-ASV | Accuracy | 0.00E+00 |
| Order-ASV | Sensitivity | 1.14E-01 |
| Order-ASV | Specificity | 0.00E+00 |
| Order-ASV | Positive predictive value | 0.00E+00 |
| Order-ASV | Negative predictive value | 0.00E+00 |
| Order-Class | AUC | 9.59E-01 |
| Order-Class | prAUC | 4.34E-02 |
| Order-Class | Accuracy | 1.00E+00 |
| Order-Class | Sensitivity | 5.27E-01 |
| Order-Class | Specificity | 7.03E-01 |
| Order-Class | Positive predictive value | 1.00E+00 |
| Order-Class | Negative predictive value | 1.00E+00 |
| Order-Family | AUC | 9.26E-01 |
| Order-Family | prAUC | 3.06E-01 |
| Order-Family | Accuracy | 2.53E-01 |
| Order-Family | Sensitivity | 9.35E-01 |
| Order-Family | Specificity | 8.51E-01 |
| Order-Family | Positive predictive value | 2.45E-01 |
| Order-Family | Negative predictive value | 6.18E-01 |
| Order-Genus | AUC | 1.53E-08 |
| Order-Genus | prAUC | 0.00E+00 |
| Order-Genus | Accuracy | 6.23E-08 |
| Order-Genus | Sensitivity | 6.16E-03 |
| Order-Genus | Specificity | 1.18E-01 |
| Order-Genus | Positive predictive value | 1.30E-07 |
| Order-Genus | Negative predictive value | 3.60E-06 |
| OTU-ASV | AUC | 3.65E-02 |
| OTU-ASV | prAUC | 7.71E-01 |
| OTU-ASV | Accuracy | 9.97E-03 |
| OTU-ASV | Sensitivity | 6.25E-02 |
| OTU-ASV | Specificity | 0.00E+00 |
| OTU-ASV | Positive predictive value | 6.99E-07 |
| OTU-ASV | Negative predictive value | 5.82E-01 |
| OTU-Class | AUC | 7.93E-07 |
| OTU-Class | prAUC | 3.41E-04 |
| OTU-Class | Accuracy | 4.87E-09 |
| OTU-Class | Sensitivity | 4.48E-03 |
| OTU-Class | Specificity | 5.43E-02 |
| OTU-Class | Positive predictive value | 1.68E-07 |
| OTU-Class | Negative predictive value | 1.55E-08 |
| OTU-Family | AUC | 1.60E-06 |
| OTU-Family | prAUC | 6.94E-06 |
| OTU-Family | Accuracy | 2.87E-04 |
| OTU-Family | Sensitivity | 2.04E-04 |
| OTU-Family | Specificity | 1.00E+00 |
| OTU-Family | Positive predictive value | 2.64E-03 |
| OTU-Family | Negative predictive value | 1.88E-04 |
| OTU-Genus | AUC | 1.00E+00 |
| OTU-Genus | prAUC | 1.00E+00 |
| OTU-Genus | Accuracy | 9.95E-01 |
| OTU-Genus | Sensitivity | 4.69E-01 |
| OTU-Genus | Specificity | 8.65E-01 |
| OTU-Genus | Positive predictive value | 1.00E+00 |
| OTU-Genus | Negative predictive value | 9.75E-01 |
| OTU-Order | AUC | 2.53E-10 |
| OTU-Order | prAUC | 0.00E+00 |
| OTU-Order | Accuracy | 0.00E+00 |
| OTU-Order | Sensitivity | 9.83E-07 |
| OTU-Order | Specificity | 8.23E-01 |
| OTU-Order | Positive predictive value | 3.35E-08 |
| OTU-Order | Negative predictive value | 2.32E-08 |
| Phylum-ASV | AUC | 4.25E-02 |
| Phylum-ASV | prAUC | 9.26E-01 |
| Phylum-ASV | Accuracy | 2.35E-03 |
| Phylum-ASV | Sensitivity | 3.41E-01 |
| Phylum-ASV | Specificity | 1.68E-08 |
| Phylum-ASV | Positive predictive value | 7.18E-08 |
| Phylum-ASV | Negative predictive value | 3.74E-01 |
| Phylum-Class | AUC | 5.95E-07 |
| Phylum-Class | prAUC | 7.11E-05 |
| Phylum-Class | Accuracy | 7.38E-08 |
| Phylum-Class | Sensitivity | 5.31E-02 |
| Phylum-Class | Specificity | 1.85E-02 |
| Phylum-Class | Positive predictive value | 1.53E-06 |
| Phylum-Class | Negative predictive value | 1.12E-07 |
| Phylum-Family | AUC | 1.21E-06 |
| Phylum-Family | prAUC | 1.11E-06 |
| Phylum-Family | Accuracy | 1.50E-03 |
| Phylum-Family | Sensitivity | 4.48E-03 |
| Phylum-Family | Specificity | 1.00E+00 |
| Phylum-Family | Positive predictive value | 1.11E-02 |
| Phylum-Family | Negative predictive value | 7.37E-04 |
| Phylum-Genus | AUC | 1.00E+00 |
| Phylum-Genus | prAUC | 1.00E+00 |
| Phylum-Genus | Accuracy | 1.00E+00 |
| Phylum-Genus | Sensitivity | 9.08E-01 |
| Phylum-Genus | Specificity | 9.70E-01 |
| Phylum-Genus | Positive predictive value | 1.00E+00 |
| Phylum-Genus | Negative predictive value | 9.98E-01 |
| Phylum-Order | AUC | 0.00E+00 |
| Phylum-Order | prAUC | 0.00E+00 |
| Phylum-Order | Accuracy | 1.20E-08 |
| Phylum-Order | Sensitivity | 4.67E-05 |
| Phylum-Order | Specificity | 6.06E-01 |
| Phylum-Order | Positive predictive value | 3.34E-07 |
| Phylum-Order | Negative predictive value | 1.59E-07 |
| Phylum-OTU | AUC | 1.00E+00 |
| Phylum-OTU | prAUC | 1.00E+00 |
| Phylum-OTU | Accuracy | 1.00E+00 |
| Phylum-OTU | Sensitivity | 9.89E-01 |
| Phylum-OTU | Specificity | 1.00E+00 |
| Phylum-OTU | Positive predictive value | 1.00E+00 |
| Phylum-OTU | Negative predictive value | 1.00E+00 |

Table S6. Tukey multiple pairwise-comparison *p*-values following one way ANOVA for all ASV, clinical variable, and *Klebsiella* genotype elastic net performance data comparisons.

| Comparison | Metric | Adjusted *p*-value |
| --- | --- | --- |
| ASV clinical genotype-ASV | AUC | 1.03E-07 |
| ASV clinical genotype-ASV | prAUC | 4.92E-10 |
| ASV clinical genotype-ASV | Accuracy | 0.001507755 |
| ASV clinical genotype-ASV | Sensitivity | 0.990179537 |
| ASV clinical genotype-ASV | Specificity | 0.001067363 |
| ASV clinical genotype-ASV | Positive predictive value | 0.00128224 |
| ASV clinical genotype-ASV | Negative predictive value | 0.115271755 |
| ASV clinical genotype-ASV clinical | AUC | 2.91E-06 |
| ASV clinical genotype-ASV clinical | prAUC | 4.59E-07 |
| ASV clinical genotype-ASV clinical | Accuracy | 6.52E-05 |
| ASV clinical genotype-ASV clinical | Sensitivity | 0.999047785 |
| ASV clinical genotype-ASV clinical | Specificity | 7.48E-06 |
| ASV clinical genotype-ASV clinical | Positive predictive value | 1.58E-05 |
| ASV clinical genotype-ASV clinical | Negative predictive value | 0.017399303 |
| ASV clinical-ASV | AUC | 0.990058478 |
| ASV clinical-ASV | prAUC | 0.534123068 |
| ASV clinical-ASV | Accuracy | 0.977628225 |
| ASV clinical-ASV | Sensitivity | 0.999895923 |
| ASV clinical-ASV | Specificity | 0.883057473 |
| ASV clinical-ASV | Positive predictive value | 0.920794598 |
| ASV clinical-ASV | Negative predictive value | 0.984634749 |
| ASV genotype-ASV | AUC | 6.31E-07 |
| ASV genotype-ASV | prAUC | 1.84E-09 |
| ASV genotype-ASV | Accuracy | 0.007244305 |
| ASV genotype-ASV | Sensitivity | 0.996968836 |
| ASV genotype-ASV | Specificity | 0.004315194 |
| ASV genotype-ASV | Positive predictive value | 0.005872862 |
| ASV genotype-ASV | Negative predictive value | 0.132690986 |
| ASV genotype-ASV clinical | AUC | 1.50E-05 |
| ASV genotype-ASV clinical | prAUC | 1.79E-05 |
| ASV genotype-ASV clinical | Accuracy | 0.000425114 |
| ASV genotype-ASV clinical | Sensitivity | 0.999895923 |
| ASV genotype-ASV clinical | Specificity | 4.42E-05 |
| ASV genotype-ASV clinical | Positive predictive value | 0.000106465 |
| ASV genotype-ASV clinical | Negative predictive value | 0.021026788 |
| ASV genotype-ASV clinical genotype | AUC | 0.999515432 |
| ASV genotype-ASV clinical genotype | prAUC | 0.980931166 |
| ASV genotype-ASV clinical genotype | Accuracy | 0.998259973 |
| ASV genotype-ASV clinical genotype | Sensitivity | 0.999993522 |
| ASV genotype-ASV clinical genotype | Specificity | 0.999149579 |
| ASV genotype-ASV clinical genotype | Positive predictive value | 0.998598077 |
| ASV genotype-ASV clinical genotype | Negative predictive value | 0.999999892 |
| clinical-ASV | AUC | 0.002606714 |
| clinical-ASV | prAUC | 0.068157216 |
| clinical-ASV | Accuracy | 0.001507755 |
| clinical-ASV | Sensitivity | 0.219633344 |
| clinical-ASV | Specificity | 0.161065192 |
| clinical-ASV | Positive predictive value | 0.00372621 |
| clinical-ASV | Negative predictive value | 0.001721346 |
| clinical-ASV clinical | AUC | 0.00021304 |
| clinical-ASV clinical | prAUC | 0.00015396 |
| clinical-ASV clinical | Accuracy | 0.020283373 |
| clinical-ASV clinical | Sensitivity | 0.131318037 |
| clinical-ASV clinical | Specificity | 0.787815984 |
| clinical-ASV clinical | Positive predictive value | 0.083189016 |
| clinical-ASV clinical | Negative predictive value | 0.018724213 |
| clinical-ASV clinical genotype | AUC | 4.79E-10 |
| clinical-ASV clinical genotype | prAUC | 4.79E-10 |
| clinical-ASV clinical genotype | Accuracy | 4.80E-10 |
| clinical-ASV clinical genotype | Sensitivity | 0.050559279 |
| clinical-ASV clinical genotype | Specificity | 5.97E-09 |
| clinical-ASV clinical genotype | Positive predictive value | 4.81E-10 |
| clinical-ASV clinical genotype | Negative predictive value | 5.20E-09 |
| clinical-ASV genotype | AUC | 4.79E-10 |
| clinical-ASV genotype | prAUC | 4.79E-10 |
| clinical-ASV genotype | Accuracy | 4.88E-10 |
| clinical-ASV genotype | Sensitivity | 0.072987305 |
| clinical-ASV genotype | Specificity | 4.89E-08 |
| clinical-ASV genotype | Positive predictive value | 5.08E-10 |
| clinical-ASV genotype | Negative predictive value | 7.28E-09 |
| genotype-ASV | AUC | 0.328087936 |
| genotype-ASV | prAUC | 4.64E-05 |
| genotype-ASV | Accuracy | 0.554709562 |
| genotype-ASV | Sensitivity | 3.42E-06 |
| genotype-ASV | Specificity | 5.43E-10 |
| genotype-ASV | Positive predictive value | 5.29E-05 |
| genotype-ASV | Negative predictive value | 0.98216709 |
| genotype-ASV clinical | AUC | 0.718741001 |
| genotype-ASV clinical | prAUC | 5.15E-09 |
| genotype-ASV clinical | Accuracy | 0.157597323 |
| genotype-ASV clinical | Sensitivity | 9.79E-07 |
| genotype-ASV clinical | Specificity | 4.79E-10 |
| genotype-ASV clinical | Positive predictive value | 3.38E-07 |
| genotype-ASV clinical | Negative predictive value | 0.999999999 |
| genotype-ASV clinical genotype | AUC | 0.001801601 |
| genotype-ASV clinical genotype | prAUC | 4.79E-10 |
| genotype-ASV clinical genotype | Accuracy | 0.224986074 |
| genotype-ASV clinical genotype | Sensitivity | 1.25E-07 |
| genotype-ASV clinical genotype | Specificity | 0.025599401 |
| genotype-ASV clinical genotype | Positive predictive value | 0.977120697 |
| genotype-ASV clinical genotype | Negative predictive value | 0.016173485 |
| genotype-ASV genotype | AUC | 0.006023082 |
| genotype-ASV genotype | prAUC | 4.79E-10 |
| genotype-ASV genotype | Accuracy | 0.461608165 |
| genotype-ASV genotype | Sensitivity | 2.67E-07 |
| genotype-ASV genotype | Specificity | 0.007779472 |
| genotype-ASV genotype | Positive predictive value | 0.863132358 |
| genotype-ASV genotype | Negative predictive value | 0.019575404 |
| genotype-clinical | AUC | 1.78E-07 |
| genotype-clinical | prAUC | 0.366984094 |
| genotype-clinical | Accuracy | 5.97E-07 |
| genotype-clinical | Sensitivity | 0.034255055 |
| genotype-clinical | Specificity | 4.79E-10 |
| genotype-clinical | Positive predictive value | 4.79E-10 |
| genotype-clinical | Negative predictive value | 0.02011976 |
