# Supplementary material for: Gut community structure as a risk factor for infection in Klebsiella pneumoniae-colonized patients
Source: mSystems. 2024 Jul 8;9(8):e00786-24. doi: 10.1128/msystems.00786-24 (PMC11334466; doi:10.1128/msystems.00786-24)
Supplement: Reviewer comments [file reviewer-comments.pdf]

AMERICAN SOCIETY FOR MICROBIOLOGY | mSystems®

Gut community structure as a risk factor for infection in Klebsiella pneumoniae-colonized patients

Jay Vornhagen, Krishna Rao, and Michael Bachman

Corresponding Author(s): Jay Vornhagen, Indiana University School of Medicine

Review Timeline:

Submission Date:	June 10, 2024
Accepted:	June 11, 2024

Editor: Nicholas Chia

Reviewer(s): The reviewers have opted to remain anonymous.

Transaction Report:

(Note: With the exception of the correction of typographical or spelling errors that could be a source of ambiguity, letters and reports are not edited. The original formatting of letters and referee reports may not be reflected in this compilation.)

DOI: https://doi.org/10.1128/msystems.00786-24

Re: mSystems00786-24 (Gut community structure as a risk factor for infection in Klebsiella pneumoniae-colonized patients)

Dear Dr. Jay Vornhagen:

Your manuscript has been accepted, and I am forwarding it to the ASM production staff for publication. Your paper will first be checked to make sure all elements meet the technical requirements. ASM staff will contact you if anything needs to be revised before copyediting and production can begin. Otherwise, you will be notified when your proofs are ready to be viewed.

Data Availability: ASM policy requires that data be available to the public upon online posting of the article, so please verify all links to sequence records, if present, and make sure that each number retrieves the full record of the data. If a new accession number is not linked or a link is broken, provide production staff with the correct URL for the record. If the accession numbers for new data are not publicly accessible before the expected online posting of the article, publication may be delayed; please contact ASM production staff immediately with the expected release date.

Publication Fees: For information on publication fees and which article types have charges, please visit our website. We have partnered with Copyright Clearance Center (CCC) to collect author charges. If fees apply to your paper, you will receive a message from no-reply@copyright.com with further instructions. For questions related to paying charges through RightsLink, please contact CCC at ASM_Support@copyright.com or toll free at +1-877-622-5543. CCC makes every attempt to respond to all emails within 24 hours.

ASM Membership: Corresponding authors may join or renew ASM membership to obtain discounts on publication fees. Need to upgrade your membership level? Please contact Customer Service at Service@asmusa.org.

PubMed Central: ASM deposits all mSystems articles in PubMed Central and international PubMed Central-like repositories immediately after publication. Thus, your article is automatically in compliance with the NIH access mandate. If your work was supported by a funding agency that has public access requirements like those of the NIH (e.g., the Wellcome Trust), you may post your article in a similar public access site, but we ask that you specify that the release date be no earlier than the date of publication on the mSystems website.

Embargo Policy: A press release may be issued as soon as the manuscript is posted on the mSystems Latest Articles webpage. The corresponding author will receive an email with the subject line "ASM Journals Author Services Notification" when the article is available online.

Cover Image Submissions: If you would like to submit a potential Cover Image, please email a file and a short legend to msystems@asmusa.org. Please note that we can only consider images that (i) the authors created or own and (ii) have not been previously published. By submitting, you agree that the image can be used under the same terms as the published article. Image File requirements: TIF/EPS, 7.5 inches wide by 8.25 inches tall (at least 2,250 pixels wide by 2,475 pixels tall), minimum 300 dpi resolution (600 dpi preferred), RGB, and no figure elements, e.g., arrows or panel labels. The legend should be a short description of the image, 1-2 sentences recommended. Please download and use this interactive template in Adobe to ensure that your proposed cover image meets our size requirements (https://journals.asm.org/pb-assets/pdf-text-excel-files/ASM-Interactive-Sizing-Cover-Template-1715689791.pdf).

Author Video:: For mSystems research articles, you are welcome to submit a short author video for your recently accepted paper. Videos are normally 1 minute long and are a great opportunity for junior authors to get greater exposure. Importantly, this video will not hold up the publication of your paper and you can submit it at any time.

Details of the video are:
· Minimum resolution of 1280 x 720
· .mov or .mp4 video format
· Provide video in the highest quality possible but do not exceed 1080p
· Provide a still/profile picture that is 640 (w) x 720 (h) max
· Provide the script that was used

We recognize that the video files can become quite large, so to avoid quality loss ASM suggests sending the video file via https://www.wetransfer.com/. When you have a final version of the video and the still ready to share, please send it to mSystems staff at mSystems@asmusa.org.

Thank you for submitting your paper to mSystems.

Sincerely,
Nicholas Chia
Editor
mSystems
